# Controls of focused fluid release in subduction zones: insights from experimental dehydration of brucite vein networks in serpentinite

**DOI:** 10.1007/s00410-025-02221-9

**Published:** 2025-04-17

**Authors:** Manuel D. Menzel, Lisa Eberhard, Austin Arias, José Alberto Padrón-Navarta, Oliver Plümper

**Affiliations:** 1 Instituto Andaluz de Ciencias de la Tierra (IACT–CSIC), Avda. de Las Palmeras no. 4, 18100 Armilla, Spain; 2 Department of Earth Sciences, Utrecht University, Princetonlaan 8a, 3584 CB Utrecht, The Netherlands; 3 Faculty of Geosciences, Bremen University, Klagenfurter Straße 2-4, 28359 Bremen, Germany

**Keywords:** Subduction metamorphism, Serpentinite dehydration, Experimental petrology, Microstructures, Deep fluids

## Abstract

Aqueous fluids released by metamorphic dehydration of serpentinites are a key component for seismicity, creep, and geochemical cycling in subduction zones. How these fluids drain and migrate towards the mantle wedge has yet to be fully understood. Here we address the influence of pre-existing structural and mineralogical heterogeneities in serpentinites on dehydration and fluid migration at forearc conditions. We partially dehydrated natural serpentinite containing brucite veins in a piston-cylinder apparatus with a temperature gradient across the conditions of the brucite + antigorite = olivine + fluid reaction (485–520 °C; 1.5 GPa). Micro-tomography, electron microscopy and microstructural analysis of the experimental results, coupled with thermodynamic modelling, show that temperature, mineralogical heterogeneity and variable ingress of external H_2_ controlled the dehydration extent. Experimentally formed olivine indicates a topotactic relationship between [100]_Ol_ and [0001]_Brc_, although the resultant fabric is overall random because brucite was randomly oriented. Olivine forms mono-mineralic aggregates along the walls of brucite veins, displaying very high porosity (up to 32%) and permeability (10^–13^–10^–14^ m^2^). Tracing the pre-existing brucite vein network, these aggregates can form a transient network of interconnected, highly permeable fluid channels that allows drainage and may enhance open-system exchange with neighboring lithologies. Infiltration of reduced external fluids can trigger redox dehydration of magnetite + antigorite to Fe-rich olivine, which renews porosity and propagates focused fluid flow. The distribution of brucite and magnetite, especially as vein networks, therefore has a first-order control on how focused fluid drainage and flow paths develop during subduction of serpentinites.

**Supplementary Information:**

The online version contains supplementary material available at 10.1007/s00410-025-02221-9.

## Introduction

The subduction of hydrous minerals is essential for Earth’s plate tectonics and the related deep geochemical and geodynamic cycles. The release of aqueous fluids from the hydrated lithosphere and subsequent fluid migration within the slab, along the plate interface and into the mantle wedge strongly influences mass transfer, seismic activity and magmatism in subduction settings. Serpentinites contain high water contents (up to 14 wt.% structurally bound H_2_O) and can be very abundant in oceanic lithosphere (e.g., Alt et al. 2013; Lissenberg et al. 2024). They are among the most important fluid sources in subduction zones, producing major fluid pulses during dehydration reactions at elevated temperatures (*T*). The reaction of brucite (Brc) + antigorite (Atg) to olivine (Ol) and aqueous fluid (hereafter referred to as “brucite-dehydration”) occurs at 480–520 °C (at ~ 1.3–4.0 GPa, respectively), which typically corresponds to forearc depths in warm and cold subduction zones. Breakdown of antigorite and chlorite occurs at higher temperatures (~ 640–670 °C, and 720–820 °C, respectively) and pressures corresponding to subarc depths. The reaction products of the latter are rarely exhumed (Padrón-Navarta et al. 2011). In contrast, serpentinites with metamorphic olivine formed after brucite dehydration are common in exhumed high-pressure (HP) metamorphic terranes (Jabaloy-Sánchez et al. 2022; Kawahara et al. 2016; Kempf et al. 2020; Peters et al. 2020; Plümper et al. 2017; Scambelluri et al. 1991; Ulrich et al. 2024). Typical structures include metamorphic olivine veins in HP-serpentinites—commonly interpreted as channelized fluid pathways—and distributed olivine throughout the matrix. Brucite dehydration has also been proposed as a trigger mechanism for intermediate depth seismicity in subduction zones (Gutiérrez-Aguilar et al. 2022), with olivine vein structures possibly being a manifestation of transient shear instabilities due to fluid overpressure (Muñoz-Montecinos et al. 2024). Thus, brucite dehydration is an ideal reaction to investigate not only the conditions controlling dehydration but also the mechanisms that determine whether fluid drainage is pervasive or channelized and the respective consequences for deformation behavior and geochemical exchange. The exact mechanisms of olivine vein formation and the extent they are coupled to deformation processes and/or reactive fluid percolation are debated. Some studies infer an important influence of compositional heterogeneities that localize dehydration and form self-organizing fluid channels due to fluid pressure gradients (Plümper et al. 2017), which may develop into larger-scale networks by reactive flow (Huber et al. 2022). Other studies indicate that stress and deformation play a major role (Jabaloy-Sánchez et al. 2022; Muñoz-Montecinos et al. 2024; Schmalholz et al. 2023). Addressing these open questions requires a better characterization of the effects of inherited chemical and structural rock heterogeneities and non-hydrostatic stresses in subducting serpentinites.

The most striking heterogeneity that may impact fluid release during brucite dehydration are veins. Brucite-serpentine veins are commonplace in abyssal, serpentinized dunites not affected by seafloor weathering (e.g., Bach et al. 2006; Klein et al. 2020), and can further form at the transition of lizardite/chrysotile to antigorite at ~ 300 °C during incipient subduction of harzburgitic serpentinites (Schwartz et al. 2013; Menzel et al. 2018). Various metamorphic Atg-serpentinites, for instance from the Sanbagawa belt, Japan (Kawahara et al. 2016; Nagaya et al. 2022), the Alps (Caurant et al. 2023; Groppo and Compagnoni 2007; Peretti et al. 1992; Schwartz et al. 2013) and the Advocate ophiolite, Newfoundland (Menzel et al. 2018) exhibit brucite veins. In the Lanzo massif, Italian Western Alps, prominent centimeter-scale brucite veins occur at the contact of Atg-serpentinite with graphitized meta-ophicalcite (Vitale Brovarone et al. 2017). Brucite veins have further been found in serpentinite clasts from mud volcanoes of the Mariana forearc (Debret et al. 2019). During progressive subduction, conditions of brucite dehydration are eventually reached and newly formed metamorphic olivine may inherit the previous vein structure without the necessity for deformation or even fracturing. This would have substantial implications for the interpretation of natural olivine vein structures as well as the mechanisms of fluid drainage and percolation. This mode of olivine vein formation has received little attention, although evidence of inherited vein structures in the form of olivine rims at the contacts between antigorite with brucite veins is found in various places (Caurant et al. 2023; Kawahara et al. 2016).

Geochemical characteristics of metamorphic olivine indicate external fluid infiltration during brucite dehydration in many HP-serpentinites (Clarke et al. 2020; Ulrich et al. 2024). Serpentinites commonly have high bulk rock Fe^3+^/Fe_total_ (Mayhew and Ellison 2020) and thus are prone to oxidize reduced components of infiltrating fluids during subduction metamorphism. Important redox reactions include the formation of olivine from magnetite + antigorite, a process that can explain the lower bulk Fe^3+^/Fe_total_ of some HP-serpentinites (Debret et al. 2015; Padrón-Navarta et al. 2023). During brucite dehydration, these redox processes significantly influence the quantities and characteristics of the released fluid. Signs of external fluid infiltration may in turn record how transient interconnected pore networks and channelized fluid flow develop at larger scales. To date, redox processes have not yet been taken into account in theoretical studies of brucite dehydration (Huber et al. 2022; Plümper et al. 2017; Schmalholz et al. 2023).

While natural field-studies provide a large-scale context, they are hampered by the superposition of various metamorphic and post-metamorphic processes and the lack of direct information about the released fluid, due to fluid drainage and compaction. Snapshots of the transient reaction stage are rarely preserved in the natural rock record. Experiments investigating the development of transient permeability during dehydration reactions in subducted serpentinite are scarce (Eberhard et al. 2022; Tenthorey and Cox 2003), and do not cover the specific conditions of brucite dehydration. In an exploratory, experimental investigation of the microstructural evolution during brucite dehydration, Nagaya et al. (2022) proposed that topotactic olivine growth on brucite plays a fundamental role during dehydration. These experiments, however, showed very limited reaction extent, and, similar to earlier experiments that were focused on phase relations and reaction rates of the MgO-SiO_2_-H_2_O system (e.g., Wegner and Ernst 1983), bear no direct insights into natural heterogeneities and the characteristics of the transient permeability that develops during dehydration.

Here, we experimentally dehydrate a brucite-rich Atg-serpentinite at P–T conditions typical for warm subduction zones. Using a sample cored from a well-characterized, natural brucite vein in an olivine-free serpentinite, we can investigate the effects of microstructural heterogeneities on the formation of olivine and related porosity. The comparatively long sample (5.5 mm cylinder length) allows us to establish a ~ 35 °C temperature gradient across the sample to study the extent of brucite dehydration as a function of temperature in a single experiment. The use of a graphite furnace causes a finite diffusion of hydrogen through the gold capsule, which allows us to further address the effects of infiltration of external reducing agents on dehydration. Using pre- and post-experiment micro-tomography (µ-CT) and electron microscopy analysis, we investigate the characteristics of olivine and porosity formed in dependence on the natural chemical and structural heterogeneity of serpentinite as well as the impact of infiltration of reduced fluids. Our results are consistent with thermodynamic models and show a very high transient permeability at brucite-vein rims, underlining the importance of pre-existing structural heterogeneities for fluid release and drainage in subducted serpentinites.

## Methods and materials

### Starting material

**Fig. 1:**
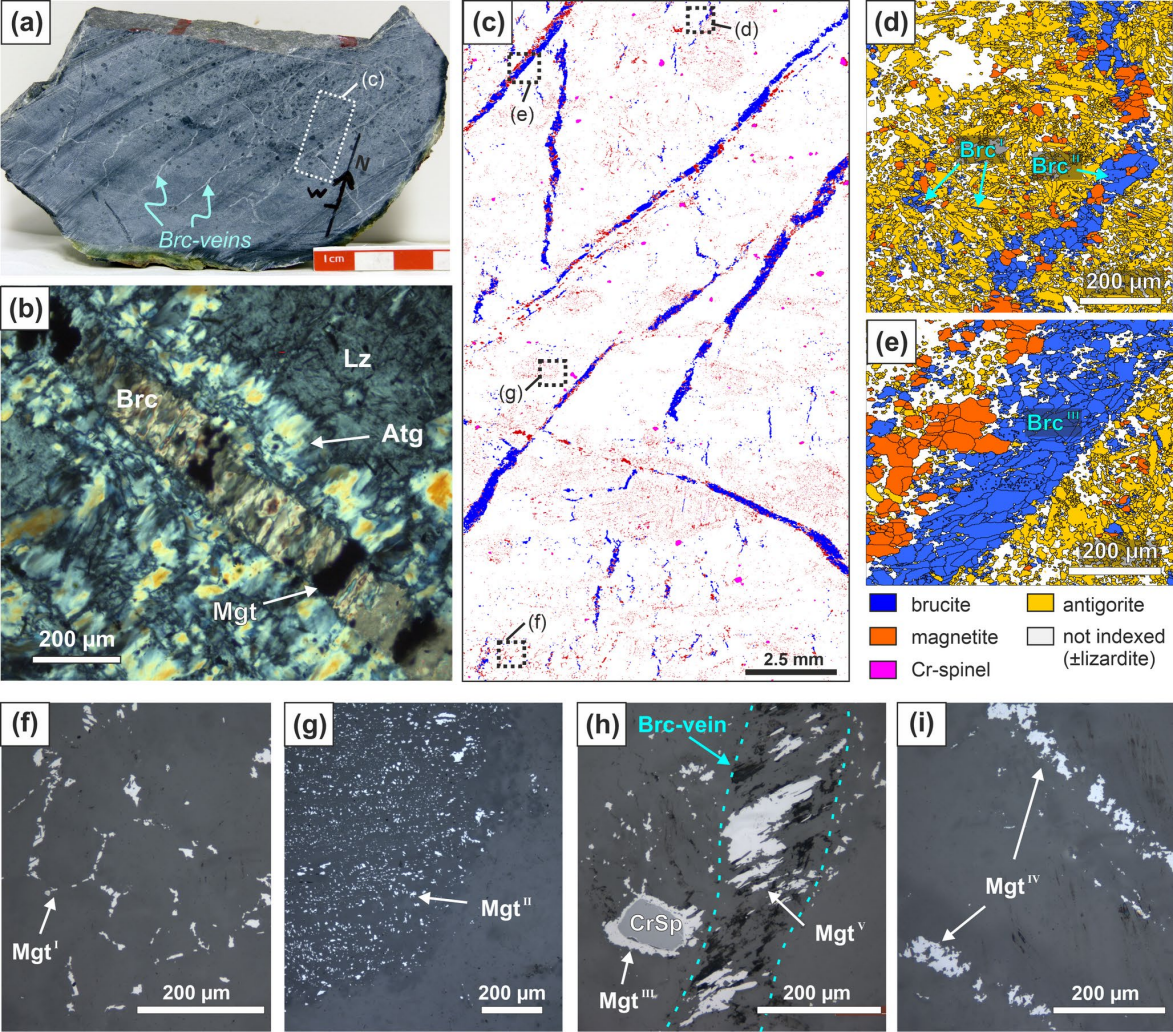
Starting material showing microstructural/mineralogical heterogeneities typical for serpentinites undergoing the lizardite to antigorite transition. **a** Slab of hand specimen, showing white conjugate brucite veins. **b** Cross-polarized light micrograph of brucite vein with palisade antigorite replacing lizardite. **c** Phase distribution of brucite (blue), magnetite (red) and Cr-spinel (magenta) from EDX mapping. **d**, **e** EBSD phase maps showing dispersed brucite (Brc^I^) and vermicular and straight veins. **f**, **i** Reflected light images of the microstructural variability of magnetite. Brc: brucite; Atg: antigorite; Lz: lizardite; Mgt: magnetite; CrSp: Cr-spinel

The starting material is a fully serpentinized harzburgite with prominent sets of brucite veins from the Advocate ophiolite complex, Newfoundland (Fig. 1). The petrography as well as the bulk and mineral chemistry of this rock are described in Menzel et al. (2018) (sample ADV-15). In the following, we summarize the most important characteristics, and add more detailed microstructural observations. The serpentinite is composed of ~ 88 wt.% serpentine, ~ 4.7 wt.% brucite, ~ 4.8 wt.% magnetite, ~ 1.1 wt.% Cr-spinel, and traces of dolomite, magnesite, pentlandite and heazlewoodite, according to bulk chemistry mass balance and petrographic analysis (Menzel et al. 2018). Olivine is not observed in this sample. The bulk rock composition is characterized by low Al_2_O_3_ and CaO contents (Supplementary Table S2), consistent with the depleted composition of the harzburgite protolith (Bédard and Escayola 2010; Menzel et al. 2018), and high Fe^3+^/Fe_total_ (0.73) and X_Mg_ = Mg/(Mg + Fe^2+^) (0.98) as expected for completely serpentinized peridotites. Serpentine (average X_Mg_ = 0.985, assuming all Fe to be Fe^2+^ in this case) is predominantly Al-poor antigorite with interlocked microstructure. Minor remnants of lizardite (Lz), particularly in bastite pseudomorphs after orthopyroxene, locally have elevated Al_2_O_3_ and Cr_2_O_3_ contents (up to 2.5 and 1.0 wt.%, respectively). Brucite has high X_Mg_ (~ 0.984) and slightly elevated MnO and NiO contents (up to 0.3 and 0.2 wt.%, respectively). Brucite occurs as small (< 30 μm) dispersed grains in the serpentine matrix (Brc^I^ in Fig. 1d), thin (20–100 μm) vermicular veinlets (Brc^II^), and as thick (0.2–4.0 mm) straight veins associated with antigorite and magnetite (Brc^III^ in Fig. 1e). Some brucite veins have shear microstructures suggesting minor deformation after their formation (Fig. 1c–e). Microstructures and compositions of brucite indicate that it formed during the prograde metamorphic transformation of lizardite to antigorite (Menzel et al. 2018). Magnetite (Mgt) occurs in various microstructural positions (Fig. 1f–i): (i) clusters of fine-grained magnetite (5–30 µm) outlining the traces of the previous lizardite mesh texture (Mgt^I^), (ii) aligned clusters of fine-grained to dusty magnetite (1–25 µm), marking bastite serpentine after previous pyroxene (Mgt^II^), (iii) magnetite rims (1 µm to up to 50 µm wide) around Cr-spinel (Mgt^III^), (iv) aggregates of moderate to coarse grained magnetite (20–150 µm) forming oxide-rich layers or veins (Mgt^IV^), and (v) variably sized magnetite grains and grain aggregates (5–200 µm) with commonly prismatic habits in brucite veins (Mgt^V^).

### High-pressure piston-cylinder experiment

**Fig. 2:**
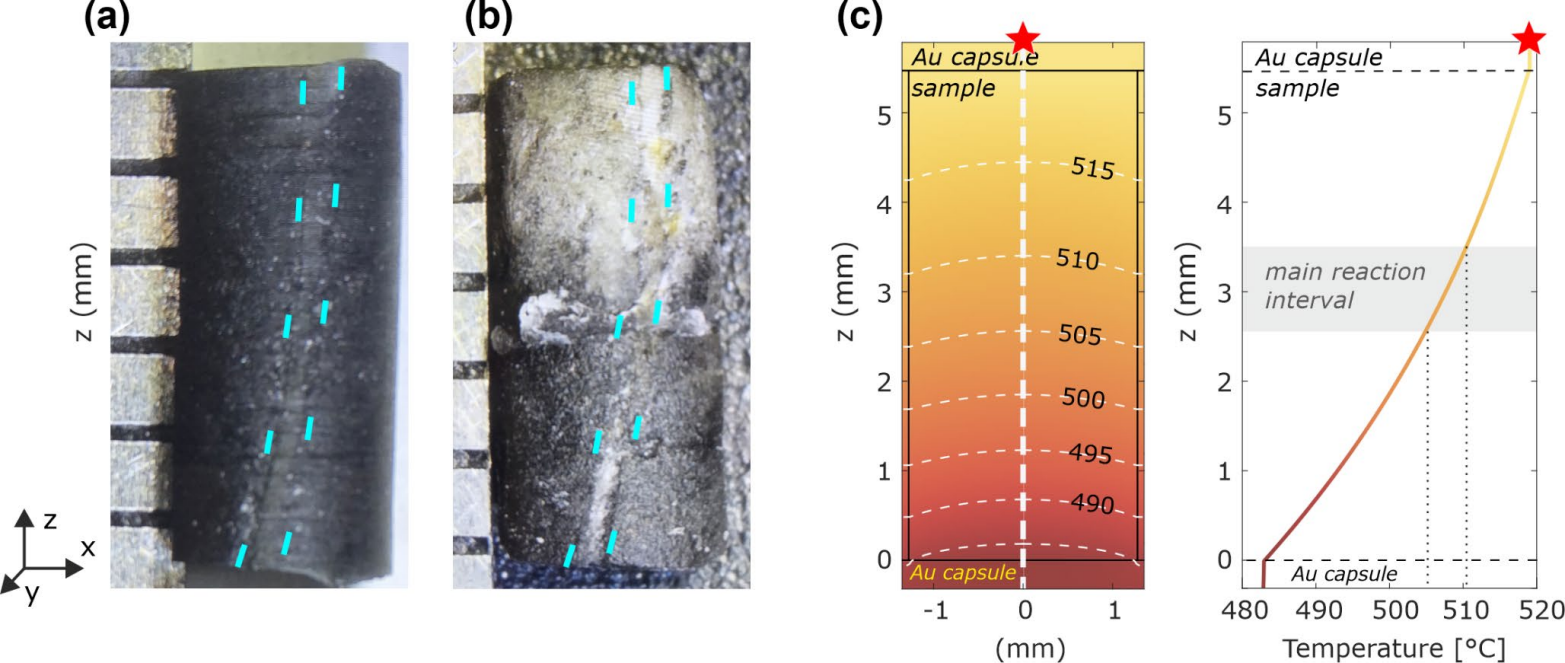
Sample cylinder (**a**) before and after (**b**) experiment, and (**c**) corresponding calculated temperature field during the experiment (white dashed contour lines and axial gradient). The main brucite vein is highlighted in a) and b) with blue dashed lines. The red star in c) indicates the position of the thermocouple. For details of the thermal modeling method and the complete temperature field of the experiment cylinder assembly see Supplement B

We prepared a cylindrical drill core of 5.5 mm length and 2.6 mm diameter obtained from a rock slab of the starting material, so that it contains a macroscopically visible brucite vein oriented subparallel with respect to the cylinder axis (Fig. 2a). The drill core was analyzed with µ-CT prior to the experiment (pre-scan). The sample was subsequently wrapped into a 0.025 mm thick Al-foil to prevent the capsule material from intruding into sample porosity, and placed into a gold capsule with one end welded shut and the other side crimped together. We used a standard half-inch piston cylinder assembly with a graphite heater at the Instituto Andaluz de Ciencias de la Tierra (IACT–CSIC), Spain. The sample position was chosen with the top end of the sample capsule close to the hottest zone in the assembly, and the sample extending into the lower half of the assembly (Fig. 2; Fig. S1; see Sect. “Modelling of the temperature field in the experiment”). In this way a monotonous and approximately uni-directional temperature gradient is achieved along the cylinder axis of the sample, which allows us to track mineral reactions and porosity generation as a function of temperature in a single experiment. The experiment was kept at 1.5 GPa and 520 °C (max. temperature at the top of the capsule) for 87 h. Temperature was measured with a type-K thermocouple, located at the top of the sample. The used assembly allows to investigate the effect of infiltration of a reducing agent, because the MgO-moisture-graphite assemblage at the heater induces H_2_ ingress into the comparatively oxidized sample (Supplement F; Fig. S14).

After quenching the sample was slowly decompressed. Nevertheless, we could not avoid a decompression crack that parted the sample in the center. After carefully scraping off all gold of the capsule, the sample was fit together and re-analyzed by µ-CT (post-scan) before further processing. The sample was then cut parallel to the core cylinder axis and perpendicular to the brucite vein orientation (x–z section, c.f. Fig. 2a), using a slow diamond wire saw, and subsequently mounted in epoxy and polished for scanning electron microscopy analysis.

### *Micro-X-ray computer tomography *(*µ-CT*)

The µ-CT pre-scan was done at the University of Granada with a Zeiss Xradia 510 X-ray µ-CT system, using 3201 projections obtained at 80 kV, 7 W and 8 s exposure time. For the post-scan we used a Zeiss Xradia 620 Versa X-ray µ-CT system at Utrecht University, acquired at 50 kV, 4.5 W and 22 s exposure time and 1601 projections. The voxel size was set to 1.35 µm for both measurements. All µ-CT scans were corrected for beam hardening and cone effects.

The µ-CT attenuation contrast between serpentine and brucite was insufficient for a reliable segmentation of both phases due to their similar densities and the strong contrast of abundant dispersed magnetite in the samples. The main brucite vein is well visible in µ-CT in the reacted sample part because it is delineated by olivine (Ol) along the rims, but brucite-serpentine contacts could not be distinguished. Therefore, we segmented the phases serpentine (includes brucite), magnetite, spinel, olivine, and air, applying a supervised machine learning workflow using the Ilastik software for pixel-segmentation with random-forest classifier accounting for texture, edges and Gaussian features (Berg et al. 2019). The machine learning model was trained on respective sub-volumes of µ-CT slices with manual phase labelling. Phase modes along the sample cylinder axis were determined by image analysis using Fiji/ImageJ2 (Schindelin et al. 2012). The final 3D visualization was generated using Dragonfly Pro. Large Cr-spinel grains with characteristic magnetite rims were used as anchor points to correlate the 3D pre- and post-scan µ-CT data with the 2D SEM–EDX mapping of the axial section of the post-experiment sample.

### *Scanning electron microscopy *(*SEM*)* analyses*

Backscattered electron (BSE), energy dispersive x-ray spectroscopy (EDX) and electron back-scatter diffraction (EBSD) maps were obtained using a Zeiss EVO^®^ MA 15 SEM equipped with an Oxford Instruments UltimMax-170 EDX and Nordlys Nano-EBSD detectors at the IACT–CSIC. Simultaneous EBSD–EDX maps were acquired with step sizes of 1–3 µm on colloidal-silica polished, non-coated samples, using standard EBSD analytical settings (see Supplement A for extended details of all SEM analyses and data processing). Data treatment of EBSD results with the Oxford HKL Channel5 software included noise reduction and minor extrapolation to fill non-indexed pixels. We further corrected systematic misindexation of pseudo-symmetries, and phase misidentification artefacts using the simultaneously acquired EDX data. In one detailed EBSD map, we divided the dataset into subsets corresponding to Mg-rich and Fe-rich olivine according to the EDX data. We used MTEX (version 5.11.2; Bachmann et al. 2010) for further analysis of the EBSD results. Grain reconstruction was done with a 10° segmentation angle, followed by the removal of small grains (≤ 8 pixel). For analysis of the crystallographic misorientations between neighboring brucite and olivine grains we performed an additional grain reconstruction step in a separate treatment so that phase boundary segments are well represented.

BSE and high-quality EDX mapping and point analysis were acquired on carbon-coated samples at high vacuum. EDX point analysis followed a standard-less approach, applying a beam current calibration on Co every 30 min. EDX mapping was obtained with a step size of ~ 400 nm and 1 ms acquisition time per pixel. We used Fiji/ImageJ2 to obtain segmented phases by image processing of combinations of individual element and element ratio maps, and for particle analysis of brucite and magnetite grains/grain aggregates. To obtain a compositional map of X_Mg_ in olivine from the EDX count intensities of Mg and Fe, we applied composition-depended correction curves derived from the spot measurements in the mapped area.

### Modelling of the temperature field in the experiment

We calculated the temperature field in the experiment (including the capsule, the assembly and the piston cylinder vessel) by adapting the numerical thermal model from Moarefvand et al. (2021), solving the heat equation. The geometry of the model setup was adjusted to the piston-cylinder apparatus used for our experiment. Volumetric and thermal parameters of all materials are implemented as T-dependent functions derived from literature data (Supplementary Table S1). The thermocouple temperature at the top of the sample capsule was used as a reference for the numerical model. We fixed the temperature at the outer boundary to 25 °C (cooling water temperature) and ran the model until the modelled temperature at the thermocouple junction converged to the experimental temperature. Model details and a list of all parameters used for the temperature calculation are given in Supplement B.

### Permeability calculation

The permeability of the newly formed mono-mineralic olivine rims along the walls of the brucite vein was derived from a 3D sub-volume of the post-experiment µ-CT data in the most reacted region (1863 × 883 × 718 µm^3^ domain from the top of the reacted sample). We evaluated the permeability of the olivine–pore aggregate using randomly sampled domains with variable volumes. The representative volume was defined as the domain volume for which the statistical properties of the assemblage converge. For each sampled domain we calculated the porosity, defined as the volume fraction of pore space, and two-point correlation functions. Permeability of each sampled domain was calculated by solving the Stokes equations along each Cartesian axis (reference frame of µCT sample as indicated in Fig. 2) and applying Darcy’s law on the homogenized average flow velocities (Torquato 2002). The resultant permeability tensors are then diagonalized using the eigenvalues of the original matrices. More details on the workflow and permeability calculation can be found in Supplement C.

### Thermodynamic modelling

To quantify the effect of chemical heterogeneities, temperature and infiltration of reducing fluid, we calculated isobaric T-X equilibrium phase diagrams using the Gibbs energy minimization software Perple_X (version 7.1.6) (Connolly 2005). The calculated thermodynamic models assess the compositional variations between brucite-free serpentinite (referred here as X_Brc_ = 0) and the brucite vein (X_Brc_ = 1), with different quantities of H_2_ added to the system (X_H2_ given in mol/kg rock, with the oxidized composition X_H2_ = 0 corresponding to the initial bulk Fe^3+^/Fe_total_ of 0.73). This approach allows to investigate the effects of different effective equilibration volumes. Modelling was done for the simplified Fe–Mg–Al–Si–H_2_–O_2_ chemical system (see Supplementary Table S2 for normalized bulk rock composition). We applied the ds6.2 thermocalc database for minerals (Holland and Powell 2011) (DEW19HP622ver_elements.dat in Perple_X) with COH-fluid modelled according to Connolly and Galvez (2018) using the fluid equation of state for H_2_O from Pitzer and Sterner (1995) and the deep earth water model for electrolytic aqueous fluid species (Huang and Sverjensky 2019). Solid solution models were used for olivine and orthopyroxene (Jennings and Holland 2015), antigorite (Eberhard et al. 2023), chlorite (White et al. 2014), and brucite and talc (ideal mixing models). For consistency with solid solutions in serpentinite, we adjusted the free energy of the ferric chlorite endmember following Evans and Frost (2021). Magnetite and iron were treated as pure phases. All thermodynamic models shown here are calculated at isobaric pressure of 1.5 GPa.

## Results

The sample recovered after the experiment shows substantial reaction in the upper half of the cylinder (white part in Fig. 2a). Numerical modelling of the temperature field in the capsule during experiment indicates a 35 °C vertical, non-linear temperature gradient from 485 °C at the bottom to nearly 520 °C at the top of the sample (Fig. 2b). Radial temperature differences are negligible (Supplementary Fig. S2). Comparing the macroscopic sample appearance with the modelled temperature field, we infer that the main transition from non-reacted to reacted meta-serpentinite in the experiment likely occurred at a temperature of 505–510 °C (Fig. 2).

### Mineral assemblages and chemistry after experiment

**Fig. 3:**
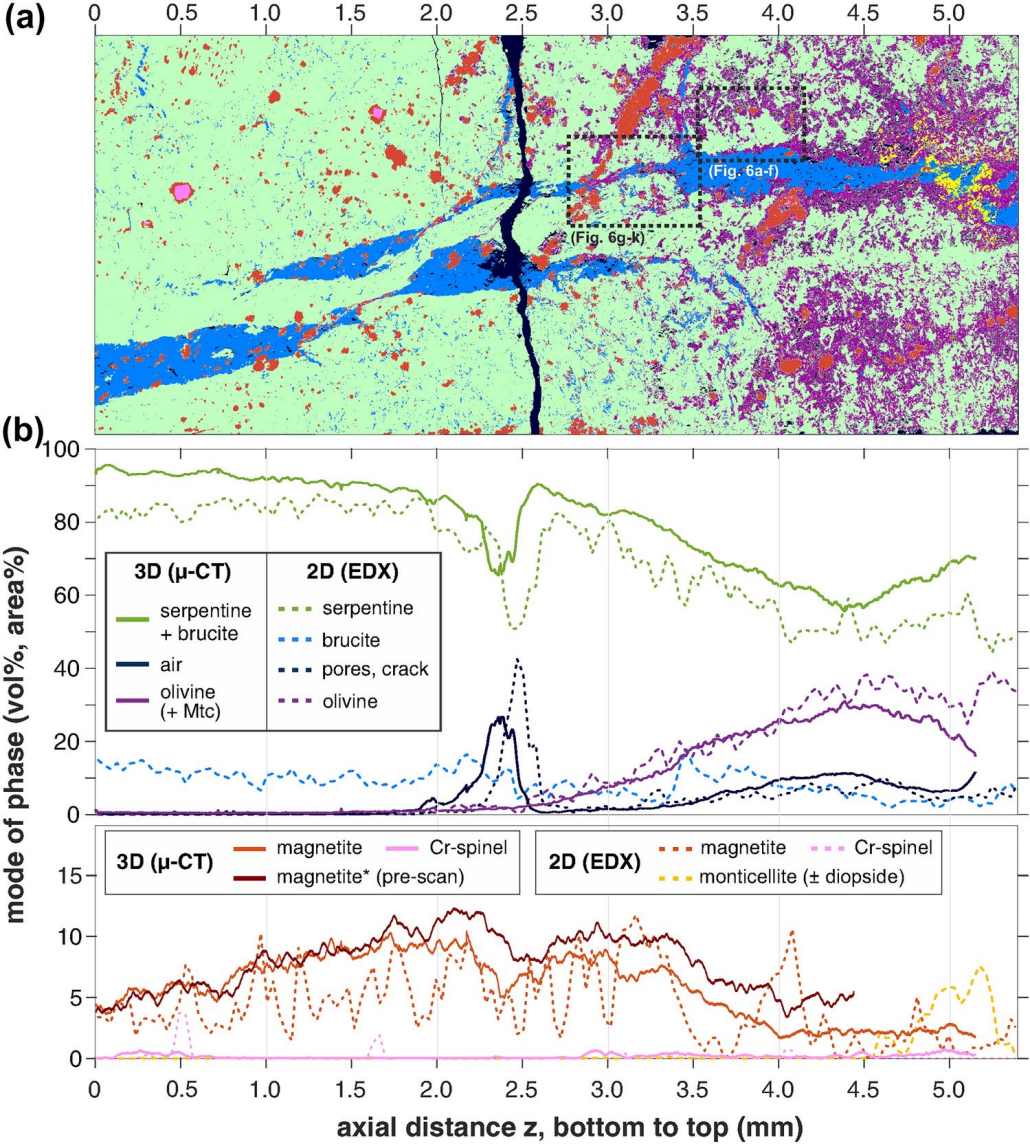
Mineral assemblages after experiment. **a** Segmented EDX phase map of the axial section of the sample recovered after experiment, and (**b**) corresponding phase proportions from µ-CT (solid lines) and EDX (dashed), averaged along z. Magnetite* is the modal abundance of magnetite from µ-CT analysis before experiment, for qualitative comparison (both pre- and post-scan magnetite abundance from µ-CT are slightly overestimated). High pore/air abundance at z = 2.4 mm corresponds to the decompression crack visible in the map. Color coding in **a** see legend in** b**

SEM and µ-CT analyses of the recovered sample reveal variable extent of metamorphic reaction from the cold end at the bottom to the hot end at the top of the sample cylinder. Only sparse reaction occurred in the bottom half, preserving the initial mineral assemblage and microstructures of the starting material (c.f. Figs. 1 and 3a). In contrast, in the top half abundant olivine and porosity formed in expense of brucite, serpentine and magnetite (Fig. 3). The modal proportions of minerals and porosity obtained from phase segmentation of the post-experiment 3D µ-CT analysis and of 2D EDX mapping in the polished section are mutually consistent and show the same averaged trends from the cold to the hot part of the sample (Fig. 3b). Olivine abundance increases up to 38 vol.% towards the hot end, related to a matching increase in porosity to up to 11 vol.% averaged across the bulk sample (not counting the decompression crack visible in Fig. 3a). Brucite remains stable also at the hot end, as inclusions in olivine and within the vein rimmed by olivine, but not in contact with serpentine. The width of olivine rims along the brucite vein walls significantly increases from 10–20 µm to > 100 µm towards the hot end. Close to the uppermost part of the sample, monticellite (Mtc; CaMgSiO_4_) locally formed within the brucite vein, never in contact with antigorite, and related to rare diopside in interstices of the antigorite–olivine matrix nearby (Fig. 3; Supplementary Fig. S16). Cr-spinel has partially hollowed-out grain shapes in the reacted part of the sample, being replaced by olivine and Cr-rich magnetite. The mode of magnetite significantly decreases in the reacted half of the sample compared to the magnetite abundance prior to experiment (Fig. 3b): fine-grained magnetite (Mgt^I^ and Mgt^II^) is completely consumed, while only remnants of larger grains and aggregates as well as magnetite enclosed within the brucite vein (Mgt^V^) remain. Rare native Fe (< 0.02 vol.%) formed at rims of magnetite grains, independently of temperature but predominantly within or in vicinity of the brucite vein and, in places, close to the rims of the sample cylinder. Similar native Fe or Fe–Ni alloys were not observed in the starting material.

**Fig. 4:**
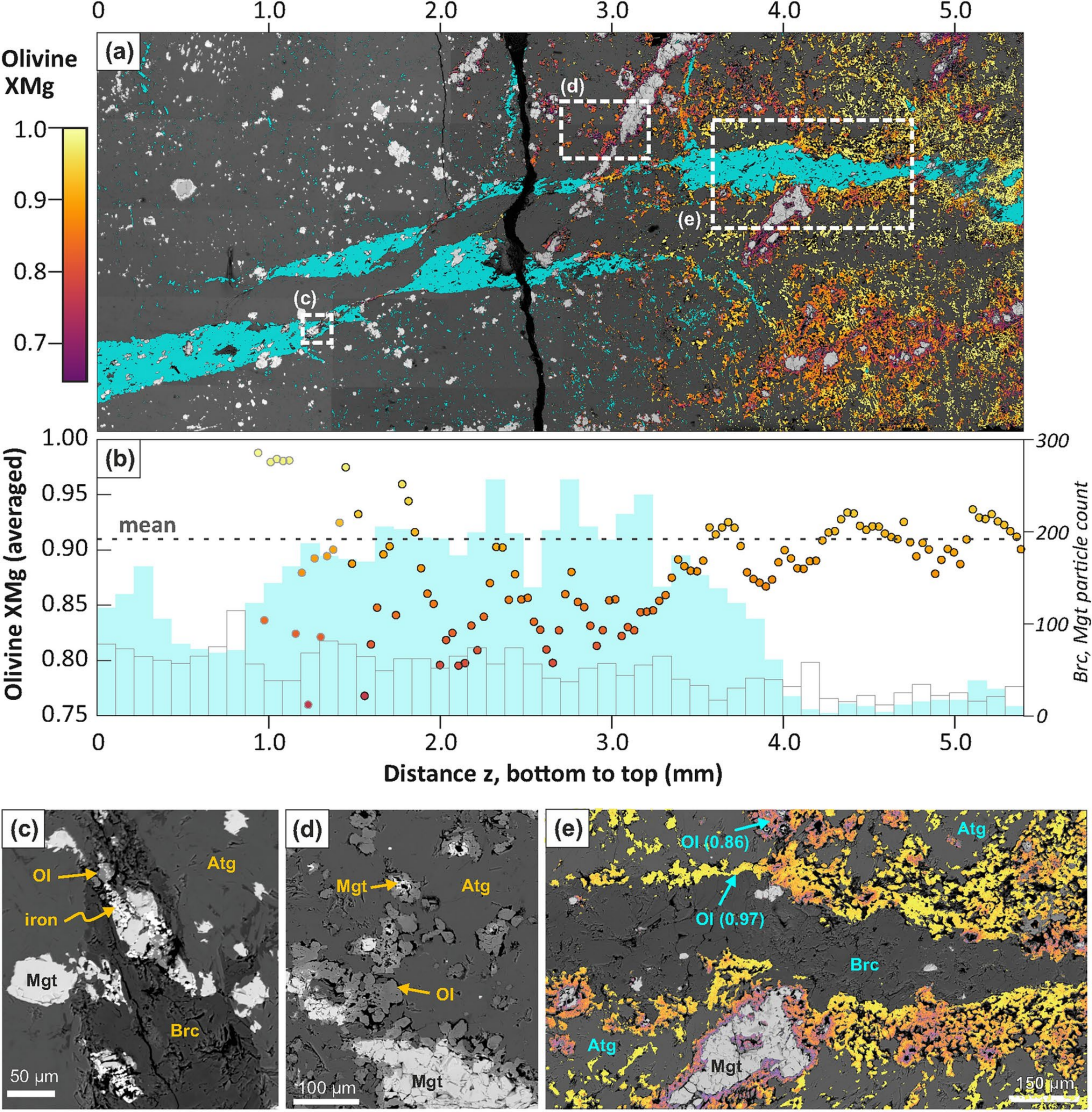
Olivine compositions and microstructures after experiment (SEM–EDX analysis). **a** Map of X_Mg_ of segmented olivine (color scale), superposed on the phase distribution of brucite (cyan) and a BSE map. **b** Plot of olivine X_Mg_ in a), averaged for 35 µm intervals of z from bottom to top (colored points). Mean X_Mg_ (dashed line) is based on the full map, excluding outlier compositions (X_Mg_ < 0.65) that are likely to be mixed magnetite-olivine analyses. Column plots show the frequency of isolated brucite (cyan) and magnetite particles (transparent). **c** First Fe-rich olivine associated with brucite, antigorite and magnetite partially replaced by native iron (z = 1.4 mm). **d** Fe-rich olivine replacing magnetite (z = 3.1 mm); olivine X_Mg_ in the shown area varies mostly between 0.75 and 0.92. **e** Detail of overlay of olivine X_Mg_ map from a) superposed on a BSE image showing porous, Mg-rich olivine reaction rims along the walls of the main brucite vein (z = 3.5–4.6 mm); X_Mg_ from EDX point measurements are given in brackets

SEM–EDX mapping shows a wide compositional range for olivine, with X_Mg_ mostly ranging from ~ 0.70 up to 0.99 in different microstructural sites (Fig. 4). Qualitatively this compositional variation is also observable in the µ-CT data. Fe-rich olivine is closely associated to remnant or previous magnetite, and generally olivine becomes more Mg-rich with increasing temperature from bottom to top. Averaged over all of the mapped area (Fig. 4a) and excluding very Fe-rich outliers (X_Mg_ < 0.65), which are likely mixed analyses at magnetite–olivine contacts, the mean X_Mg_ of olivine is ~ 0.91. In the lower part of the sample, at corresponding low temperatures, olivine occurs as rare, small grains (< 10 µm) along thin fractures parallel to the brucite vein walls and along pre-existing shear planes that transpose the brucite vein in the bottom third of the sample (z = 1.2–2.1 mm). This olivine is commonly rich in Fe (X_Mg_ ≈ 0.7–0.8) and associated to magnetite, native iron, brucite and antigorite (Fig. 4c). With increasing temperature towards the upper part of the sample (z > 2.1 mm), abundant Fe-rich olivine aggregates appear surrounding magnetite grains, associated with significant porosity preserved at olivine–serpentine contacts (Fig. 4d). These olivine aggregates show gradually decreasing Fe contents from the contacts with magnetite (X_Mg_ as low as 0.7) to the contact with antigorite (X_Mg_ up to 0.97) (Fig. 4e). In places the olivine aggregates enclose larger pores with hollowed magnetite remnants, or cavities where small magnetite grains were present before (Fig. 4d). In the sample interval from z = 3–4 mm, the frequency of olivine with X_Mg_ > 0.95 substantially increases. This correlates with the disappearance of dispersed brucite grains in the serpentinite matrix (Fig. 4a, b). In the uppermost part of the sample (z = 4.0–5.4 mm), olivine in contact with the brucite vein has high X_Mg_ (yellow colors in Fig. 4e)—as expected from the high X_Mg_ of brucite in the starting material—while Fe-enriched olivine marks the former presence of magnetite. Gradual compositional changes between Mg- and Fe-rich olivine are, however, common. The X_Mg_ of antigorite and remnant brucite remain unchanged compared to the starting material.

### Microstructures and texture

**Fig. 5:**
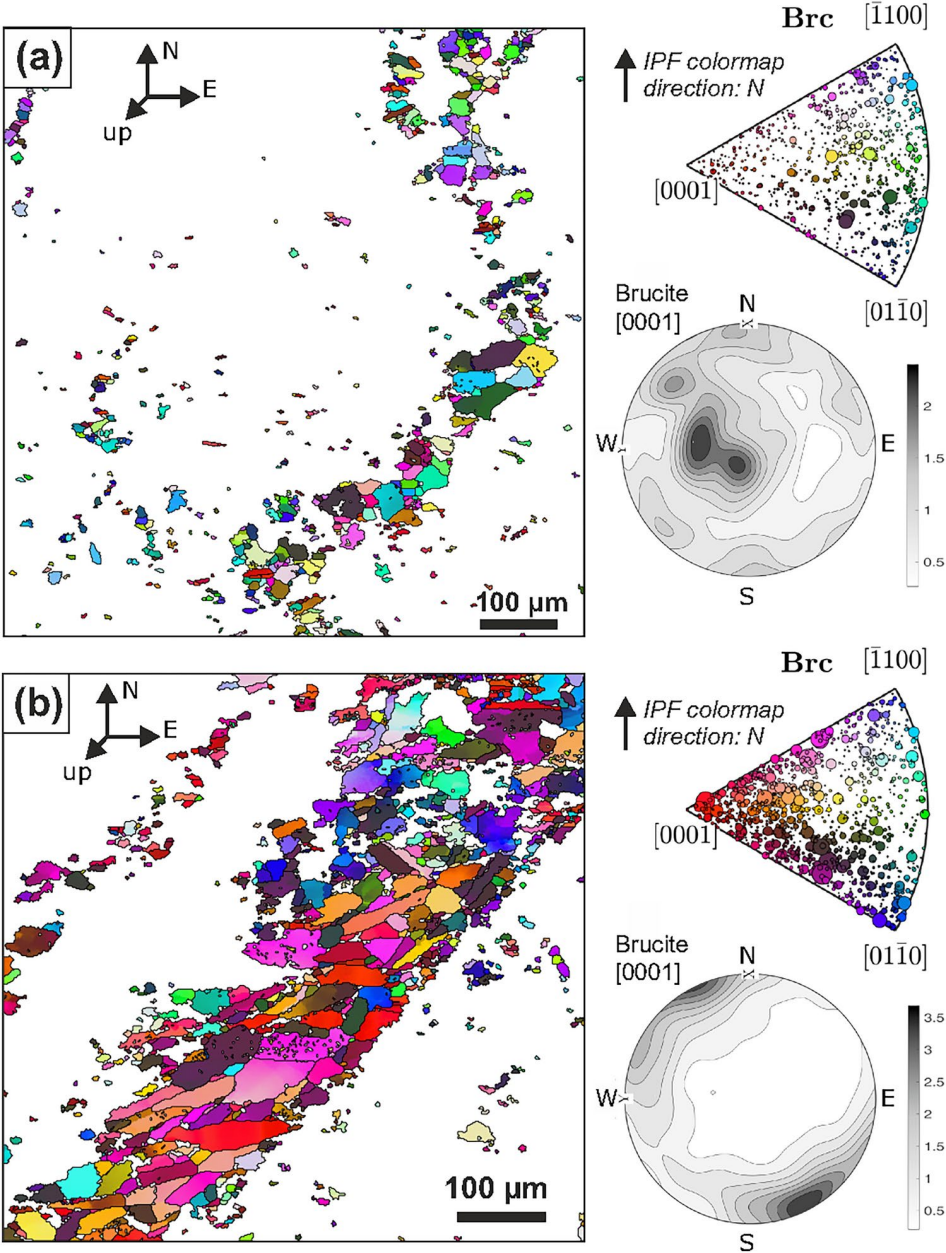
Crystallographic orientations of brucite in the starting material: EBSD orientation maps and corresponding inverse pole figures and contoured pole figures of the orientation distribution function of (**a**) dispersed brucite and vermicular vein (phase map see Fig. 1d) and (**b**) of a wide, straight brucite vein (c.f. Fig. 1e). Orientations are plotted with the inverse pole figure (ipf) color-scheme in direction of geographic N, with point sizes in inverse pole figures proportional to grainsize. Contoured pole figures of [0001]_Brc_ are plotted in lower hemisphere as multiples of a uniform distribution

The serpentinite used here as starting material (Fig. 1) is massive, without well-developed macroscopic foliation or related shape and crystallographic preferred orientations (SPO and CPO, respectively) of antigorite and brucite. EBSD analysis shows that at the microscale of single veins, brucite locally has moderately strong SPO and CPO (Fig. 5a, b). However, these vary substantially in their strength and orientation for different veins.

**Fig. 6:**
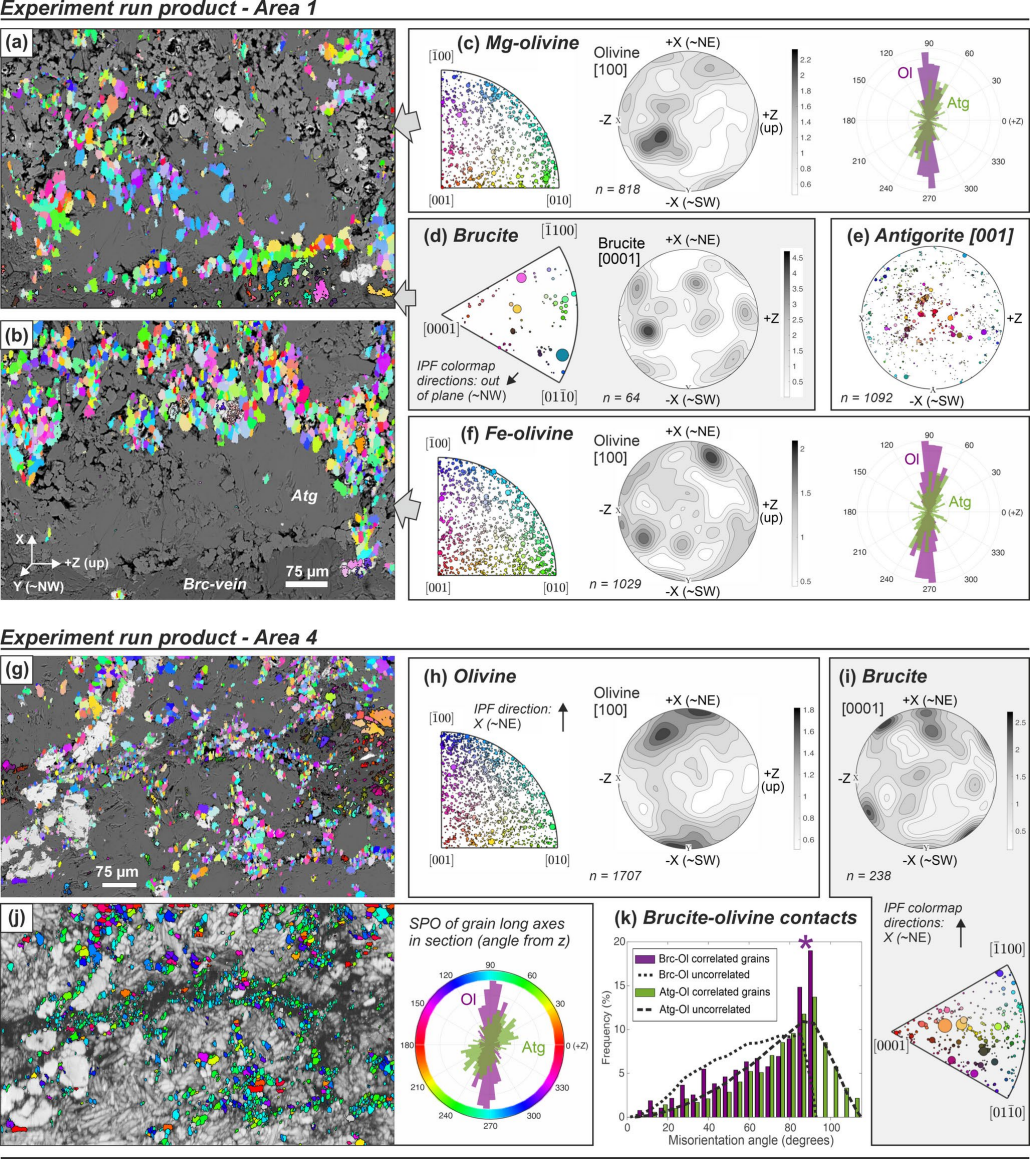
Crystallographic and shape orientations of olivine formed in experiment. EBSD orientation maps of (**a**) Mg-rich olivine and brucite (the latter highlighted with black grain boundaries) and (**b**) Fe-rich olivine (without grain-boundaries) and magnetite (with black grain-boundaries), superposed on the BSE-image of the area. **c–f** Corresponding inverse pole figures, contoured pole figures of the orientation distribution function, and orientation distribution of grain long axes of Mg-olivine, brucite, antigorite and Fe-olivine. **g–i** Orientation map and pole figures of olivine and brucite in a different area of the experimental sample. **j** Olivine in the same area as g) colored corresponding to the orientation of long axes of grains, superposed on the EBSD band contrast, and corresponding distribution showing diverging SPO for olivine and antigorite. **k** Distribution of misorientation angles of correlated (neighboring) and uncorrelated (random) grains for brucite-olivine and antigorite-olivine, showing a preference of 90° angles for neighboring brucite-olivine grains (purple star). Orientations are plotted with the inverse pole figure (ipf) color-scheme in the indicated direction, respectively, with point sizes in inverse pole figures proportional to grainsize. Spatial reference frame is indicated in **b**. Contoured pole figures are plotted in lower hemisphere as multiples of a uniform distribution

Olivine formed in the experiment does not have a clear overall CPO, showing an apparent random fabric (Fig. 6). However, at the local scale, weak but statistically significant and consistent trends in crystallographic orientations of olivine in comparison to those of remnant brucite are evident. In particular Mg-rich olivine grains exhibit weak CPO with [100]_Ol_ close to [0001]_Brc_ (Fig. 6c, d). This relationship is consistently present in different measured areas, even though the local orientations of the [100]_Ol_ and [0001]_Brc_ vary in different domains (Fig. 6a–i). Neighboring olivine and brucite grains have a preference for 90° misorientation angles at phase boundaries (Fig. 6k), consistent with a local parallelism of brucite [0001] and olivine [100]. Olivine and antigorite grains do not show any special relationship as misorientation angles of non-correlated (not-neighboring) and neighboring grain pairs of both phases display the same distribution.

Olivine grains analyzed by EBSD in the axial section have a weak but persistent SPO (mean aspect ratio ~ 1.7 after correcting for electron beam drift effects), with the long-axis preferentially oriented horizontally with respect to the sample cylinder and normal to the main brucite vein (Fig. 6j). The observed SPO is similar for Mg-rich and Fe-rich olivine, and independent of crystallographic orientation, i.e., similar across different areas of the sample.

### Pore structure and permeability

**Fig. 7:**
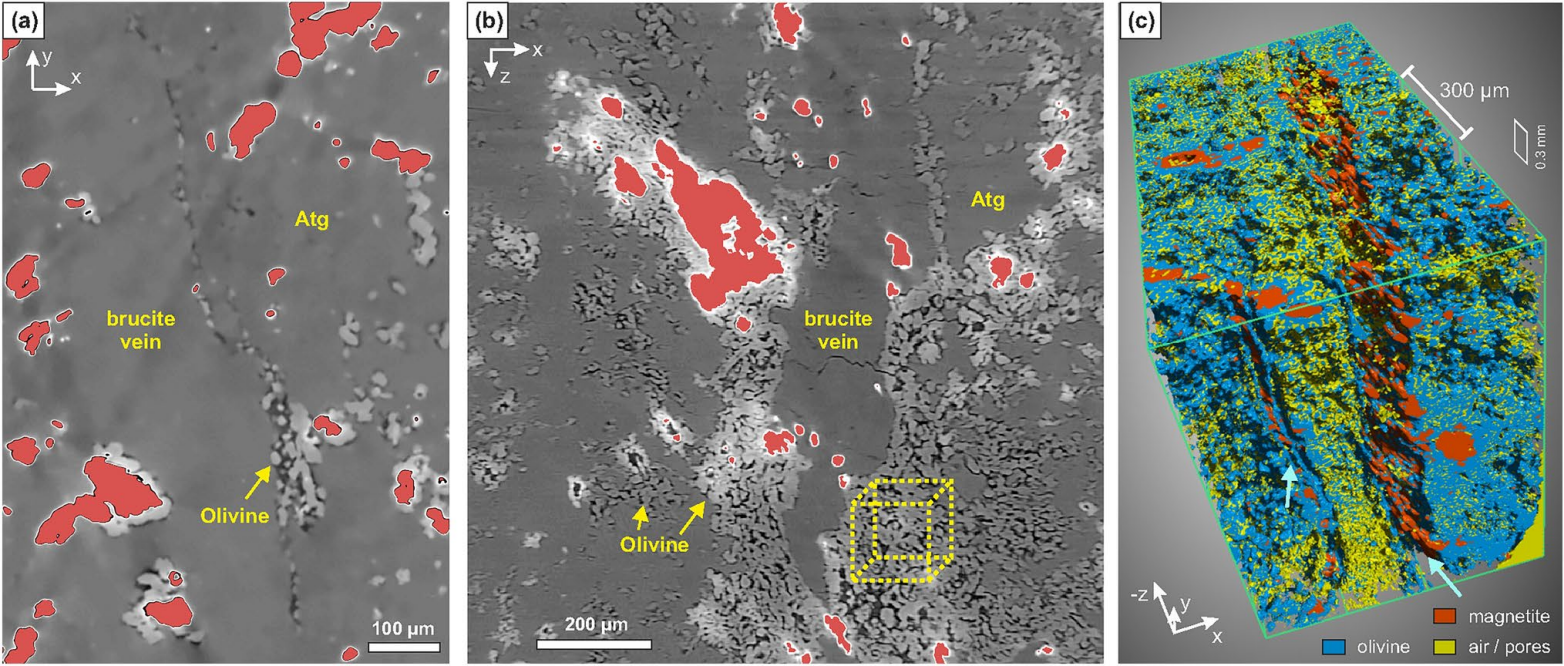
Porosity microstructures from µ-CT analysis. **a** Porosity related to incipient olivine formation along the brucite vein rim (radial section at z ≈ 2.6 mm; magnetite (red) was masked for enhancement of contrast. Smeared black stripes extending from magnetite are residual artefacts after beam hardening correction). **b** Porosity in olivine-rich domains at the brucite vein rims in the upper part of the sample. Higher grey values of olivine correspond to Fe-enriched compositions (c.f. Fig. 4e) (axial section from z ≈ 3.6 mm at the top to z ≈ 4.8 mm at the bottom of the image; magnetite masked as in **a**. The yellow cube schematically shows an example of a representative olivine-pore aggregate volume similar to those used to calculate the effective permeability in these domains (see text and Supplement C for details). **c** 3D rendering of segmented olivine, magnetite and air/pores in a subvolume in the most reacted, uppermost part of the sample. The location of the main brucite vein is correlated with the domain with high proportion of prismatic magnetite; a smaller parallel brucite vein rimmed by olivine is visible the left (see arrows)

The post-experiment µ-CT analysis shows that much of the porosity formed by dehydration remained open during and after experiment, although some compaction during the experiment as well as pore widening during decompression is likely. Apart from decompression fractures and pores within the remnant brucite vein, nearly all porosity in the reacted part of the sample is closely correlated with the distribution of olivine (Fig. 7). Pore sizes also correlate with local olivine abundance: pores in olivine-rich domains can be > 10 µm in diameter, whereas pores in antigorite-rich domains with few olivine grains are usually < 5 µm wide. Pore shapes are commonly elongated to vermicular, following the outlines of olivine grains. At the transition from the non-reacted to reacted part of the sample, thin fractures along and parallel to the brucite vein wall are evident in the µ-CT data (Fig. 7a). In places, small olivine grains are present along these thin fractures. Particularly porous domains occur in the mono-mineralic olivine rims along the walls of the brucite vein (up to 30% porosity on a 100–200 µm scale; Fig. 7b). Porosity in such olivine–pore aggregates generally shows high connectivity. Through randomized sampling of µ-CT sub-volumes of these olivine–pore aggregates, we obtain a permeability of 5 × 10^–14^ to 1 × 10^–13^ m^2^ (c.f. Supplement C). The values show a small variability between principal directions, with a slightly lower permeability in direction perpendicular to the main brucite vein. This is supported also by statistical measures, such as the two-point correlation function, that suggest a structural anisotropy in X direction (Supplementary Fig. S7). Nonetheless, the anisotropy is comparatively small, suggesting that overall, the permeability in the olivine-pore aggregates is approximately isotropic.

### Modelled phase relations

**Fig. 8:**
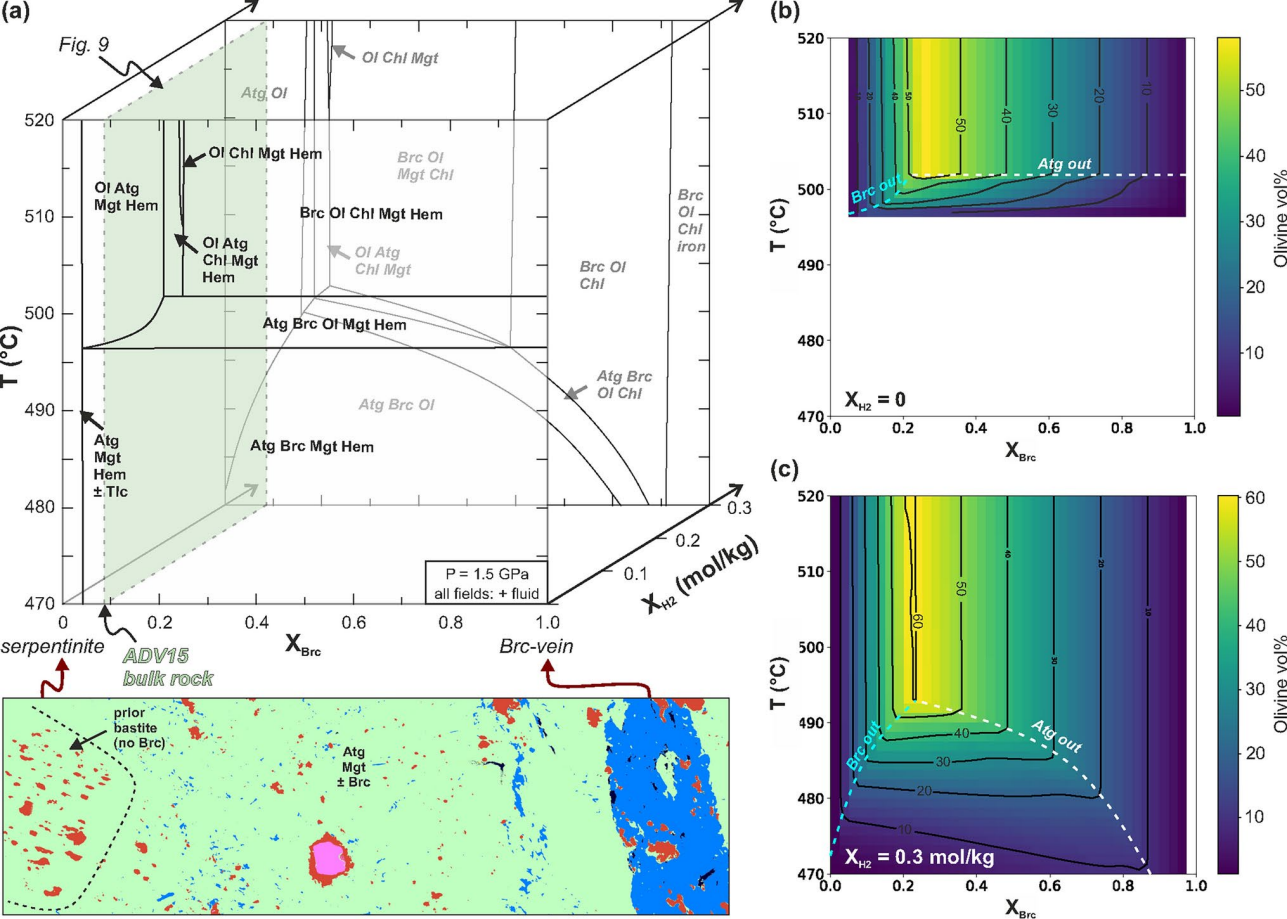
Thermodynamic model of brucite-serpentinite dehydration in dependence of temperature, brucite-serpentine proportion (X_Brc_), and reduction by hydrogen infiltration (X_H2_). **a***T*-X_Brc_ phase diagrams for X_H2_ = 0 (black labels) and X_H2_ = 0.3 mol/kg (grey italic labels), illustrating the role of the local effective bulk composition on the temperature of the reaction brucite + antigorite = olivine. The X_Brc_ axis corresponds to the compositional variation from brucite-free serpentinite to magnetite-bearing brucite vein interior as observed in different local domains of the starting material (here illustrated by the phase map of the non-reacted sample part; c.f. Fig. 3a). **b**, **c** Volume proportions of olivine for X_H2_ = 0 and 0.3, respectively, relative to the assemblage including COH-fluid. Plots of phase modes and mineral X_Mg_ for these and further X_H2_ sections are included in Supplement D, Figs. S11, S12

**Fig. 9:**
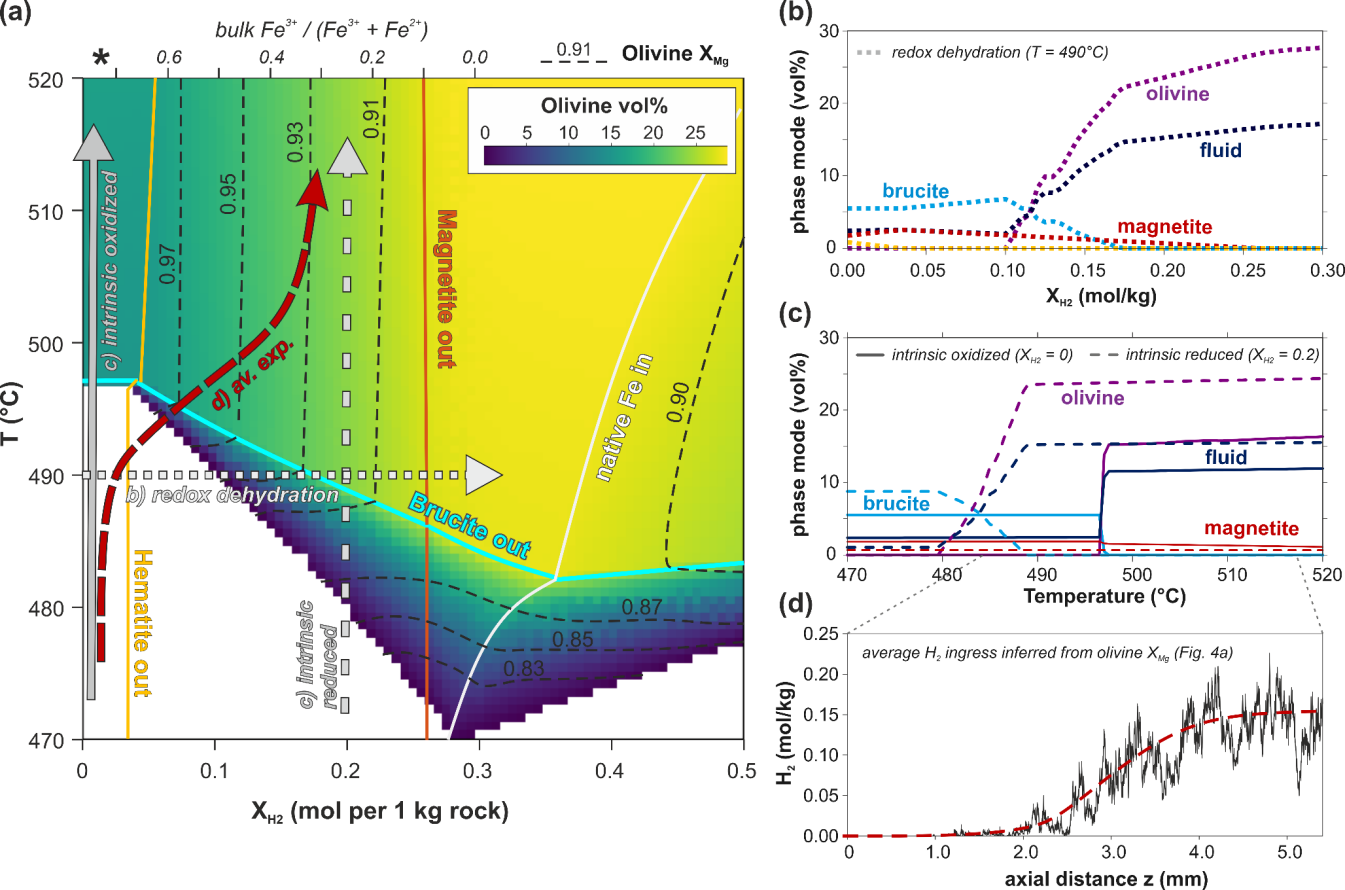
Thermodynamic model of brucite dehydration in dependence of temperature and X_H2_ for the whole rock composition of the starting material serpentinite. **a***T*-X_H2_ phase diagram, showing the stability limits for hematite, brucite, magnetite and native iron, the olivine volume abundance (colormap) and olivine X_Mg_ composition (contour lines). The equivalent bulk Fe^3+^/Fe_total_ is shown on the upper on the upper axis, with that of the starting material indicated by *. **b, c** Evolution of phase modes along three exemplary dehydration paths (dotted, dashed and filled grey arrows in **a**. **d** Average H_2_ ingress along z of the experimental sample inferred from olivine X_Mg_ (see Supplement E for calculation details), normalized to the same units as X_H2_ shown in the phase diagram. Correlation of z with the thermal gradient (c.f. Fig. 2) then provides the averaged *T*-X_H2_ path observed in experiment (red long-dashed arrow shown in **a**)

Figure 8 shows isobaric equilibrium phase diagrams as a function of (i) temperature, corresponding to the temperature gradient in the experiment capsule, (ii) the compositional variability between brucite-free serpentinite and the interior of brucite veins (X_Brc_ = 0.0 and 1.0 in Fig. 8, respectively), and (iii) different quantities of H_2_ added to the chemical system (X_H2_). The latter reflects hydrogen ingress into the capsule due to the lower oxygen fugacity at the graphite furnace compared to the reacting serpentinite (Supplementary F), analogous to open-system infiltration of reduced fluids in nature. Figure 9 shows the effect of gradual H_2_ ingress at a bulk composition of X_Brc_ ~ 0.09 (Fig. 8), corresponding to the whole rock composition of the serpentinized harzburgite starting material (Supplementary Table S2).

Oxidized bulk compositions without brucite (X_Brc_ = 0, X_H2_ = 0 in Fig. 8) do not show metamorphic reactions in the investigated temperature range. Brucite is predicted to fully dehydrate at 497–503 °C for effective bulk compositions containing up to 21 vol.% brucite (X_Brc_ up to ≈ 0.22). Rock domains corresponding to hydrated dunite (X_Brc_ ≈ 0.23) are expected to form very olivine-rich assemblages with up to 57 vol.% olivine (Fig. 8b), 34 vol.% aqueous fluid and minor chlorite, magnetite and hematite. Rock domains with compositions with > 23 vol.% brucite (X_Brc_ > 0.25) are expected to preserve brucite beyond 503 °C due to complete consumption of antigorite (Brc–Ol–Chl–Mgt–Hem assemblage in Fig. 8). Pure brucite domains (X_Brc_ = 1.0 in Fig. 8), corresponding to the inside of veins, are not predicted to dehydrate since the brucite–periclase reaction occurs at much higher temperatures (~ 900 °C at 1.5 GPa; Mirwald 2005).

Open-system ingress of H_2_ shifts all dehydration reactions to lower temperatures, both in brucite and antigorite deficient rock compositions (e.g., phase diagram at X_H2_ = 0.3 mol/kg in Fig. 8; and T–X_H2_ section in Fig. 9). The onset of brucite dehydration is lowered by up to 25 °C because Fe-rich olivine is stabilized at the expense of magnetite, antigorite and brucite (olivine with X_Mg_ < 0.89 at brucite-stable conditions, Fig. 9a). Olivine X_Mg_ generally decreases with H_2_ ingress, although this effect is partly compensated by increasing olivine abundance during brucite dehydration (Fig. 9). For the whole rock composition of the starting material, magnetite is expected to disappear after ~ 0.27 mol H_2_ ingress per kg rock, and native iron becomes stable at high X_H2_ (Fig. 9). In brucite-rich domains (X_Brc_ > 0.9 in Fig. 8), native iron already becomes stable at < 0.3 mol/kg H_2_ ingress because less Fe-olivine can form where antigorite is scarce. In contrast, serpentinite domains locally enriched in magnetite (c.f. Fig. 1; not modelled here) require more H_2_ ingress for complete magnetite consumption.

## Discussion

### Factors controlling dehydration: temperature and chemical heterogeneity

Three main factors control the fluid release from brucite dehydration in subducted serpentinites: (i) temperature, (ii) the chemical composition in the effective equilibration volume, which is controlled by the micro-structural variability of the rock, and (iii) potential infiltration of reducing external fluids. Our experimental results record the effects of all three variables, documenting dehydration across a range of temperatures for variable compositional domains and different extent of reduction by H_2_ ingress. The latter is due to the very low oxygen fugacity imposed by the pressure medium and the graphite heater, inducing H_2_ ingress via diffusion and possibly through the crimped part of the capsule (Supplement F and Fig. S14; see also Golubkova et al. 2016). The formation of olivine in our experiment results from a combination of two end-member chemical reactions—the dehydration of nearly pure brucite and antigorite (mean X_Mg_ ≈ 0.984 and 0.985, respectively) to Mg-rich olivine and H_2_O:

$$ \begin{aligned} {2}0{\text{ Mg}}\left( {{\text{OH}}} \right)_{{2}} + {\text{ Mg}}_{{{48}}} {\text{Si}}_{{{34}}} {\text{O}}_{{{85}}} \left( {{\text{OH}}} \right)_{{{62}}} \\ = {\text{34 Mg}}_{{2}} {\text{SiO}}_{{4}} + {\text{ 51H}}_{{2}} {\text{O}} \end{aligned}$$

R1 $$ brucite + antigorite = olivine + fluid $$

and the reaction of magnetite, antigorite and H_2_ to Fe-rich olivine and H_2_O (“redox-dehydration”):

$$\begin{aligned} {\text{Mg}}_{{{48}}} {\text{Si}}_{{{34}}} {\text{O}}_{{{85}}} \left( {{\text{OH}}} \right)_{{{62}}} + { 6}.{\text{67 Fe}}^{{{2} + }} {\text{Fe}}^{{{3} + }}_{{2}} {\text{O}}_{{4}} + { 6}.{\text{67 H}}_{{2}} \\ = {\text{34 Mg}}_{{{1}.{41}}} {\text{Fe}}_{{0.{59}}} {\text{SiO}}_{{4}} + { 37}.{\text{67 H}}_{{2}} {\text{O}} \end{aligned}$$

R2 $$ antigorite \, + \, magnetite \, + \, H_{2} = \, Fe{\text{-}}rich \, olivine \, + \, fluid $$

The amount of reaction products depends on the local brucite–antigorite proportions and H_2_ ingress (Fig. 8). According to the thermodynamic models, the highest temperature at which brucite and antigorite in direct contact are stable is 502 °C for oxidized bulk compositions at 1.5 GPa (Fig. 8b), and decreases to ≥ 482 °C at more reducing conditions (Fig. 9a). In our experiment, the maximum temperature of preserved antigorite-brucite contacts is ~ 513 °C (c.f. Figs. 2, 3, 4a). The lowest temperature at which olivine formed around magnetite is ~ 497 °C (z = 1.2 mm in Figs. 2, 3, 4a). In between, a snapshot of the transient reaction stage is preserved in the middle part of the sample cylinder. The temperatures of the onset of olivine formation and the maximum brucite–antigorite stability inferred from the experiment are about 10–15 °C higher compared to the thermodynamic predictions (Fig. 8). Considering the inherent uncertainties of the thermocouple measurement in piston-cylinder experiments (~ 10 °C), of the calculation of the thermal gradient (c.f. Supplement B) and of the thermodynamic data, in addition to expected slow reaction kinetics close the reaction boundaries, the consistency between experiment (Figs. 3, 4) and thermodynamic models (Fig. 8) is remarkably high.

The onset of reaction R2 in experiment, manifested by the formation of Fe-rich olivine rims around magnetite and the disappearance of small-grained magnetite, occurred at about 5 °C less than the onset of R1 (c.f. Figs. 2, 4). Similarly, remnant magnetite grains are already entirely surrounded by Fe-rich olivine coronas where brucite-antigorite contacts are still observable (z = 3.1–3.9 mm). This relative temperature difference between the onset of R1 and R2, as well as the distribution and composition of reaction products in the experiment, are consistent with the prediction of the redox-sensitive thermodynamic models, showing that reducing agents lower the dehydration temperature (Figs. 3, 4, 8, 9).

Within the temperature range of 470–495 °C, redox dehydration leading to consumption of magnetite can occur at isothermal conditions, solely by infiltration of a reducing agent (Fig. 9b). This causes a more gradual transition compared to intrinsic (closed system), oxidized brucite dehydration. A similar dehydration behavior may occur in serpentinite rock domains that had previously been reduced (intrinsic reduced dehydration path in Fig. 9c). However, the starting material has high bulk rock Fe^3+^/Fe_total_ (*in Fig. 9a)—a common characteristic of fully hydrated peridotites (Debret et al. 2024; Mayhew and Ellison 2020; Padrón-Navarta et al. 2023)—showing that the experiment is an analogue to reduced fluid infiltration during brucite dehydration.

The reduction of magnetite to Fe^2+^ in olivine by H_2_ ingress can be simplified as:

R3 $$ {\text{Fe}}^{{{2} + }} {\text{Fe}}^{{{3} + }}_{{2}} {\text{O}}_{{4}} + {\text{ H}}_{{2}} = {\text{ 3 Fe}}^{{{2} + }} {\text{O}}_{{({\text{Olivine}})}} + {\text{ H}}_{{2}} {\text{O}}. $$

We are thus able to constrain the minimum amount of H_2_ infiltrated into the sample based on the Fe-content in olivine (Fig. 4), while accounting for Fe contained in antigorite and brucite (section “starting material”) to exclude olivine formed from redox-neutral brucite dehydration (Supplement E; Fig. S13). Averaging the H_2_ influx as a function of z (Fig. 9d) and the related temperature gradient (Fig. 2b), we obtain the approximate T-X_H2_ path observed in the experiment (red long-dashed path in Fig. 9a). This trend is non-linear, with a steeper increase at the temperatures of the dehydration front, indicating that H_2_ ingress was likely not only controlled by temperature but enhanced by the fluid-filled, interconnected porosity formed upon dehydration. This is supported by the observation of redox dehydration and consumption of magnetite occurring throughout all of the interior of the reacted sample part, and not only at the outer rims of the sample cylinder. Nonetheless, local H_2_ consumption by redox dehydration was heterogeneous (Fig. 9d), in places reaching complete magnetite consumption. This underlines the strong influence of compositional and microstructural heterogeneity caused by the various textural appearances of magnetite (Fig. 1).

### Controls of olivine growth and pore structure: microstructures and stress

Besides temperature and chemical heterogeneity, the microstructures—i.e. the distribution, grain sizes, SPO and CPO—of brucite, antigorite, magnetite, and of the newly formed olivine affect fluid release during brucite dehydration because they result in structural anisotropies of the porosity network.

The crystallographic orientation of brucite may strongly influence nucleation and growth mechanisms of olivine if a topotactic relationship between both minerals exists (Nagaya et al. 2022). Our results show indications for a weak but consistent correlation between [0001] of brucite and [100] of Mg-rich olivine that becomes evident as local CPO patterns and through a preference of 90° misorientation angles of neighboring grains (Fig. 6). Kikuchi patterns of low quality are prone to misindexation between brucite and olivine because the primary bands of [0001]_Brc_ and [100]_Ol_ are very similar (Supplementary Fig. S15). Correcting the EBSD data based on simultaneous EDX acquisition and filtering of small grains, as applied here, can reduce these artefacts. We infer that the parallelism of [0001]_Brc_ and [100]_Ol_ is likely a topotactic relationship, because Mg-rich layers in (0001) of brucite are parallel to octahedral-rich layers in (100) of olivine. Furthermore, the spacing of Mg positions forming the hexagonal symmetry around [0001] in brucite (0.314 nm) is very similar to the spacing between Mg positions that form the hexagonal pseudo-symmetry around [100] in olivine (0.299–0.321 nm; Supplementary Fig. S15). The crystallographic fit may lower the nucleation energy of olivine, and consequently lead to a statistically significant preference between [0001]_Brc_ and [100]_Ol_ of neighboring grains, consistent with the EBSD data from our experiment. Although different to that proposed by Nagaya et al. (2022), we consider this topotactic relationship as more robust given the much higher number of analyzed grains and the first-order crystallographic fit. Thus, assuming that brucite develops a CPO with (0001)_Brc_ parallel to (001)_Atg_ in foliated Atg-serpentinites, olivine formed by brucite dehydration may show a fabric with a preferred orientation of a-axes perpendicular to the foliation plane.

However, the effect of topotaxy on the resulting olivine fabric during brucite dehydration may commonly be weak, if not insignificant. Brucite in our starting material does not show a strong and consistent CPO (Fig. 5)—thus, olivine crystallographic orientations are expected to be overall random even though topotaxy may be important at grain-to-grain contacts (Fig. 6). Furthermore, the energetic preference of topotaxy may easily be outweighed by other effects, such as deviatoric stress. This inference is in line with observations for antigorite dehydration to olivine–enstatite at higher temperature: static experiments indicated a similar topotactic relationship between [001]_Atg_ and [100]_Ol_ (Padrón-Navarta et al. 2015), consistent with the similar lattice spacings of Mg positions in the respective planes (Fig. S15). In contrast, natural crystallographic fabrics of antigorite dehydration commonly point to important effects of stress, deformation and/or fluid migration instead (Padrón-Navarta et al. 2015). Liu et al. (2024) recently drew similar conclusions for antigorite dehydration to talc–olivine.

The shapes of olivine grains and grain aggregates are likely more important for the resulting porosity structure and permeability than the crystallographic fabric. The SPO of olivine in the experiment was not controlled by the crystallography, because it is consistent between different sample domains whereas the CPO patterns show substantial local variation. In the absence of significant deformation, the SPO must have formed by directed grain growth, possibly due to a combination of (i) chemical gradients and related gradients in saturation states, (ii) a partial, anisotropic spatial confinement, and (iii) deviatoric stress:

i. The local chemical gradients between brucite and the antigorite matrix are the main driving forces for olivine nucleation and growth during brucite dehydration at static conditions. Nonetheless, the observed olivine SPO (Fig. 6) cannot be explained solely by the orientation of local chemical gradients, since radial distributions of the long axes of elongated olivine grains around individual brucite and magnetite grains in the rock matrix are not consistently observed.
ii. Anisotropic spatial confinement of an SPO of the antigorite matrix may affect olivine growth. While the SPO of antigorite, together with the grain shape of pre-existing brucite, has a clear influence on the shape of olivine aggregates, the SPO of single olivine grains in the experiment does not always coincide with that of antigorite (Fig. 6j). Spatial confinement further occurs when high olivine nucleation rates at brucite vein walls lead to rapid contact of olivine grains such that growth is only possible in direction normal to the vein wall. If growth competition is not strongly favoring one crystallographic orientation, this can cause palisade vein microstructures (e.g., Dunkel et al. 2017). The observed olivine SPO at brucite vein rims has some reminiscences of palisade growth (Fig. 6j); however, the observed SPO is not restricted to vein walls.
iii. Deviatoric stress influences ripening and growth, because the preferential dissolution of unfavorably oriented nuclei and grains may produce anisotropic shape fabrics and potentially also crystallographic fabrics. Thus, olivine produced by brucite dehydration may show similar effects of deviatoric stress as those observed for antigorite dehydration to Chl-harzburgite (Dilissen et al. 2018) or talc-olivine fels (Clément et al. 2019), and during the dehydration of gypsum to bassanite (Gilgannon et al. 2023). Although nominally static, sample capsules in piston-cylinder experiments may experience deviatoric stress due to the applied axial force. Furthermore, at the experiment P–T conditions, brucite dehydration (R1) is volume increasing and, similar to other dehydration reactions, can cause fluid overpressures if fluid is not drained (e.g., Connolly 1997; Eberhard et al. 2022; Padrón-Navarta et al. 2011; Skarbek and Rempel 2016). Thus, volume expansion in the upper part of the capsule (Figs. 2, 3) coupled with high fluid pressure gradients, likely contributed to an axial deviatoric stress. The SPO of the long axes of olivine grains oriented perpendicular to Z may thus be due to stress-controlled grain growth. While porosity in our experiment mostly remained intact, in nature the rate of fluid drainage can additionally influence olivine grain shapes as sudden fluid-pressure drops can lead to surface-energy controlled anisotropic growth (Dilissen et al. 2021).

### Processes causing heterogeneities in serpentinites before and during incipient subduction

Our results show that chemical and structural heterogeneities are key factors influencing fluid release during brucite dehydration. Such heterogeneities, with spatially variable brucite and magnetite distribution, are commonplace in subducting serpentinites due to various processes acting during the evolution from serpentinization to incipient subduction.

**Fig. 10:**
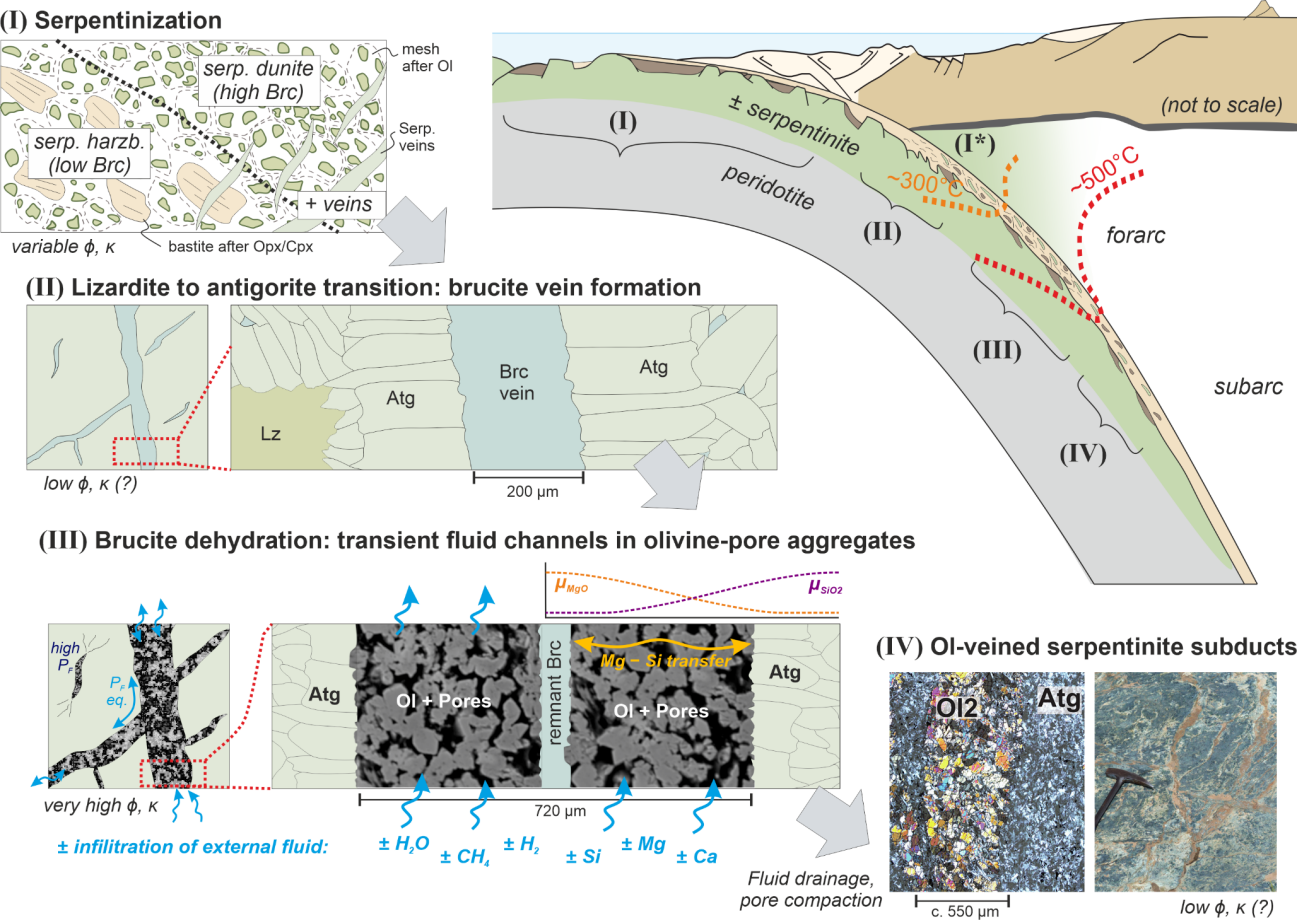
Sketch of the evolution of brucite-bearing serpentinites during subduction, illustrating structural heterogeneities formed during (I) serpentinization and (II) the lizardite to antigorite transition, and (III) their implications during brucite dehydration at forearc conditions. While isolated dehydrated brucite tension gashes may show high fluid pressure (*P*_F_) and stress concentrations, olivine-pore aggregates formed along brucite vein networks could easily interconnect and equilibrate pore pressures over longer distances, potentially allowing external fluid infiltration. Ultimately, serpentinite containing olivine vein networks subduct further (IV). The widths of the initial brucite veins are arbitrary, while those of formed olivine–pore aggregates are shown in the corresponding proportions expected from thermodynamic prediction. The micrograph in (IV) is from an olivine vein formed by brucite dehydration from Zermatt, Central Alps (adapted from Ulrich et al. 2024); the field image shows an olivine vein network in H*P*-serpentinite from Almirez, S-Spain (c.f. Jabaloy-Sánchez et al. 2022)

At decimeter to meter scales, primary chemical variations such as intercalations of lherzolite, harzburgite and dunite are the dominant heterogeneities: dunitic protoliths can lead to high brucite content during serpentinization, whereas brucite will be mostly absent in serpentinized lherzolites. The degree of serpentinization and secondary fluid-rock interactions (e.g., Klein et al. 2020) are other considerable large-scale heterogeneities affecting brucite abundance. However, here we focus on microscale textures and heterogeneities since these critically influence fluid flow during brucite dehydration. Serpentinites commonly preserve inherited microscale heterogeneities in form of pseudomorphs with bastite and mesh microstructures after pyroxene and olivine, respectively (Fig. 10). Brucite formed during serpentinization commonly occurs as dispersed and intergrown grains between serpentine mesh cores and together with magnetite and serpentine along mesh veins (Bach et al. 2006; Schwarzenbach et al. 2016). Further heterogeneities are serpentine and/or magnetite veins produced by serpentinization or in response to fracturing and dissolution–precipitation in serpentinite (Andreani et al. 2007; Tarling et al. 2021). Brucite formed during oceanic and low-T serpentinization is commonly enriched in Fe (Klein et al. 2014); similarly, low-temperature lizardite can contain significant Fe^3+^ (e.g., Mayhew and Ellison 2020).

During the low-grade metamorphic evolution towards conditions of the subduction zone forearc, additional Mg-rich brucite is expected to form through two main processes. Re-equilibration of Fe-bearing brucite and lizardite produces Mg-brucite and new magnetite, for example through the reaction:

$$ \begin{aligned} & {\text{2 Fe}}\left( {{\text{OH}}} \right)_{{2}} + { 2 }\left( {{\text{Mg}}_{{2}} {\text{Fe}}^{{{3} + }} } \right){\text{Fe}}^{{{3} + }} {\text{SiO}}_{{5}} \left( {{\text{OH}}} \right)_{{4}} \\ & \quad = {\text{ Mg}}_{{3}} {\text{Si}}_{{2}} {\text{O}}_{{5}} \left( {{\text{OH}}} \right)_{{4}} + {\text{ Mg}}\left( {{\text{OH}}} \right)_{{2}} + {\text{ 2 Fe}}^{{{2} + }} {\text{Fe}}^{{{3} + }}_{{2}} {\text{O}}_{{4}} + {\text{3 H}}_{{2}} {\text{O}} \end{aligned}$$

R4 $$ \begin{aligned} &Fe{\text{-}}brucite + Fe^{3+}{\text{-}}bearing \, lizardite \\ & \quad = Mg{\text{-}}lizardite + Mg{\text{-}}brucite + magnetite. \end{aligned}$$

Reaction R4 is formulated with Mg-cronstedtite endmember, although Fe^3+^-substitution in lizardite can also be coupled to Al-Tschermak exchange (Eberhard et al. 2023) or to vacancies (Evans et al. 2017). Recrystallization of Fe-brucite to Mg-brucite and magnetite may also be related to H_2_ release (Carlin et al. 2024; Debret et al. 2019). Metamorphic brucite further forms in closed systems during thermal breakdown of lizardite to antigorite at about 300–350 °C (Evans 2010; Schwartz et al. 2013):

$$ {\text{17Mg}}_{{3}} {\text{Si}}_{{2}} {\text{O}}_{{5}} \left( {{\text{OH}}} \right)_{{4}} \, = \,{\text{Mg}}_{{{48}}} {\text{Si}}_{{{34}}} {\text{O}}_{{{85}}} \left( {{\text{OH}}} \right)_{{{62}}} \, + \,{3} {\text{Mg}}\left( {{\text{OH}}} \right)_{{2}}. $$

R5 $$ Lizardite \, = \, antigorite \, + \, brucite. $$

Reactions R4 and R5 may occur simultaneously, explaining the high X_Mg_ of brucite and antigorite (> 0.98) as well as the intergrown prismatic habit of Mgt^V^ grains in brucite veins in our starting material. Reaction R5 significantly increases the abundance of brucite compared to serpentinization alone. Based on thermodynamic modelling and mass balance in a closed system, the brucite content in a completely serpentinized harzburgite, like our starting material, should increase from ~ 1.8 vol.% in lizardite-serpentinite to > 5.5 vol.% once all lizardite is transformed to antigorite. Si-bearing metasomatic slab fluids can prevent or consume metamorphic brucite (e.g., Schwartz et al. 2013). Nonetheless, R5 is not a dehydration reaction, hence it does not inherently produce porosity for external fluid infiltration unless other processes —like deformation— enhance permeability. The conjugate orientations of the brucite vein sets in the Advocate ophiolite demonstrate that they are related to brittle fracture, while antigorite palisade growth along the vein rims shows that the veins formed through reaction R5 (Fig. 1; and Menzel et al. 2018). Remarkably, brucite was preserved at this site, despite fracturing and nearby metasomatic fluids producing strongly carbonated serpentinites (Menzel et al. 2018), pointing to a low permeability during reaction R5. Similar preserved brucite veins are also known from other HP-serpentinites (Caurant et al. 2023; Debret et al. 2019; Kawahara et al. 2016; Nagaya et al. 2022; Schwartz et al. 2013).

Thus, brucite vein networks formed during early stages of subduction are important heterogeneities that will then influence fluid production and migration pathways during brucite dehydration at forearc depths (Fig. 10). Magnetite veins and aggregates, formed during serpentinization or by recrystallization during metamorphism, represent similar heterogeneities (Fig. 1).

### Implications for fluid release and transport mechanisms in subduction zones

Local variations of the brucite-antigorite proportions as well as of the redox budget (most importantly the Fe^3+^/Fe_total_ of the local bulk composition) in serpentinites cause spatially heterogeneous rates and quantities of fluid production at the same temperature (c.f. Figs. 8, 9). Since fluid production rates from dehydration of brucite (Liu et al. 2017) and antigorite (Chollet et al. 2011) are likely commonly faster than compaction rates by visco-elastic deformation, this favors the development of substantial fluid pressure gradients at the microscale. Such gradients will increase pore connectivity and thus enhance diffusion and advective flow (Huber et al. 2022; Plümper et al. 2017). These effects are particularly pronounced along the walls of brucite veins, because ideal proportions of antigorite and brucite lead to monomineralic olivine rims that extend in layers parallel to veins. Associated with up to 32% porosity, these olivine rims are localized fluid pathways with very high transient permeability (up to 1 × 10^–13^ m^2^; c.f. Supplement C). This permeability is several orders of magnitude higher than permeability inferred in non-dehydrated serpentinites (10^–19^–10^–24^ m^2^; e.g., Ganzhorn et al. 2019; Katayama et al. 2020; Kawano et al. 2011). Our permeability estimate is also higher than the transient permeability created during the dehydration of antigorite to olivine + talc at lower pressures (Tenthorey and Cox 2003). The main reason for these differences is that our approach allowed us to infer the transient permeability in the specific microstructural sites of high-porosity fluid channels formed by brucite dehydration rather than that of the bulk rock. Importantly, while our experiment focused on one major brucite vein, in natural serpentinite these may form conjugate networks (Fig. 1). At the mesoscale, permeability is thus mainly a function of the structure and connectivity of the pre-existing brucite vein network (Figs. 1, 10). Thus, serpentinite containing brucite veins are structurally pre-conditioned to form interconnected, highly permeable fluid channel networks during brucite dehydration (Fig. 10). This will enhance (i) the internal chemical exchange of soluble elements, (ii) potential open-system mass exchange by infiltration of external fluids, and (iii) fluid drainage.

The formation of an interconnected porosity favors element redistribution, since the chemical potentials of MgO and SiO_2_ differ significantly between olivine-brucite vein assemblages (e.g. X_Brc_ > 0.24 in Fig. 8), and the brucite-free, olivine-bearing serpentinite matrix (Fig. 10). Depending on the compaction rates, fluid-mediated diffusion along these gradients can drive Mg/Si redistribution between different dehydrated rock domains, widening the olivine aggregates at brucite vein rims while consuming remnant brucite that is otherwise shielded from antigorite. Incidentally, the distribution of local monticellite (vein center, low µ_SiO2_) and diopside (around vein rims, high µ_SiO2_) in one part of our experiment (Fig. 3a; Supplementary Fig. S16)—likely formed due to former presence of dolomite, clinopyroxene or andradite—closely reflects the expected local chemical gradients around the brucite vein. Our experimental result preserves a snapshot of the non-compacted, transient state during and directly after brucite dehydration reaction. How long such a transient state favorable for fluid-mediated diffusion and fluid flow can prevail in natural systems is unclear; this depends on the relative rates of reaction, deformation and fluid flow, in addition to the modes of fluid drainage and compaction (e.g., Bucher 1998; Tenthorey and Cox 2003). A high degree of remnant mineralogical and chemical heterogeneities in Atg-serpentinites after brucite dehydration (e.g., brucite relicts in olivine, spatially variable olivine X_Mg_; Fig. 3; see also Cacciari et al. 2025; Caurant et al. 2023) could indicate comparatively short time and length scales of equilibration, possibly due to fast compaction rates after reaction. In contrast, homogenized olivine X_Mg_ and absence of brucite inclusions may point to long equilibration time and length scales during brucite dehydration.

As long as the olivine-pore aggregates are not fully compacted, the transient network of focused fluid pathways can favor infiltration of externally derived reduced fluids, which in turn triggers further dehydration (Fig. 9). In various examples of HP-serpentinites, metamorphic olivine inferred to have formed during brucite dehydration shows geochemical and/or isotopic evidence of external fluid infiltration (Clarke et al. 2020; Ulrich et al. 2024). Besides H_2_, which can for example form by interaction of Fe–Ni alloys in serpentinites with COHS-fluids (Peretti et al. 1992) or due to high-pressure serpentinization (Vitale-Brovarone et al. 2017), a common reducing agent at forearc depths of subduction zones are CH_4_-bearing fluids derived from graphite-bearing meta-sediments (Peverelli et al. 2025; Vitale Brovarone et al. 2020). Compared to H_2_ (reaction R3), CH_4_ has a two to four times higher reduction potential, producing graphite or CO_2_, respectively. Thus, already small quantities of infiltrated fluid may have a large effect. Infiltration-driven redox dehydration of magnetite + antigorite to Fe-bearing olivine locally increases the proportion of fluid-filled porosity, creating a positive feedback that can enhance pore connectivity and thereby allow further infiltration of reduced fluid. Fluid flow will thus be guided by the presence of former magnetite veins and aggregates, in addition to brucite veins. Loss or gain of dissolved Mg or Si during reactive percolation of external fluids along the high-permeability channels (Fig. 10) may then further contribute to the forward propagation and growth of olivine veins (Huber et al. 2022; Plümper et al. 2017).

Complete fluid drainage and porosity compaction will finally leave a network of monomineralic olivine veins that are up to three times wider than the previous brucite veins (Fig. 10). This mode of fluid drainage along high-permeability fluid channels also has important mechanical implications. Dehydration of planar veins is different from that of dispersed, isolated brucite grains (Schmalholz et al. 2023), thereby influencing whether and how brucite dehydration is related to intermediate depth earthquakes or slow slip events in subduction zones (Ferrand 2019; Gutiérrez-Aguilar et al. 2022; Muñoz-Montecinos et al. 2024). Our experimentally derived permeability in the olivine–pore aggregates is well within the range inferred for episodic tremor and slip (Behr and Bürgmann 2021) and that required for related porosity wave trains (Skarbek and Rempel 2016), suggesting that inherited structural-chemical heterogeneities can play a fundamental role for drainage and flow of dehydration fluids in subduction zones. Although olivine veins in subducted Atg-serpentinites can form through various other mechanisms, the possible inheritance of previous structural heterogeneities like brucite veins has usually not been considered. We propose that this mode of olivine vein formation, potentially accompanied by infiltration-driven redox dehydration, is a common process in subducted brucite-bearing serpentinites at forearc depths.

## Conclusions

- Brucite veins formed during the lizardite–antigorite transition, together with other structural heterogeneities in serpentinites, fundamentally influence fluid release mechanisms during dehydration at forearc depths.
- Olivine formed in our experiment shows indications for a topotactic relationship with parallelism of [100]_Ol_ and [0001]_Brc_, consistent with first-order crystallographic considerations. While theoretically this may lead to an olivine fabric with preferred a-axes orientations normal to foliation, the effect of topotaxy is likely minor or even negligible in nature: (i) brucite may commonly show random orientations to begin with, and (ii) other effects influencing olivine nucleation and growth—e.g., deviatoric stress, surfactants, the speed of drainage— may strongly diminish the effect of topotaxy.
- Thermodynamic modelling and the observed H_2_ ingress and related formation of Fe-rich olivine in the experiment show that infiltration of reducing agents can trigger incipient dehydration at conditions prior to the destabilization of brucite–antigorite in oxidized serpentinites (ΔT up to 20 °C).
- Micro-CT analysis of the experimental result demonstrates that mono-mineralic olivine aggregates forming along the walls of brucite veins are domains with very high transient porosity (up to 32%) and permeability (10^–13^–10^–14^ m^2^).
- These aggregates follow pre-existing brucite veins and thus can form an interconnected network of highly permeable fluid channels. During the transient stage after equilibration of fluid overpressure and before compaction reduces porosity, these channels may allow open-system exchange with external fluids from neighboring lithologies. If infiltrated fluids contain H_2_ or CH_4_, the redox dehydration of magnetite–antigorite to Fe-bearing olivine will release additional fluid, creating a self-enhancing feedback that renews porosity and thus aids to sustain focused fluid flow.
- The distribution and grain properties of brucite and magnetite—especially when present as veins or vein networks—therefore have a first-order control on how focused fluid drainage and flow paths develop during subduction metamorphism of serpentinites. Although olivine veins in H*P*-serpentinites can also form differently, likely many indeed trace previous brucite veins. Fluid flow at depth is thus preconditioned by the structures developed during the early prograde evolution.

## Supplementary Information

Below is the link to the electronic supplementary material.

Supplementary file1 (PDF 9267 KB)

## Data Availability

Supplementary material to this article contains (A) extended SEM and EBSD analytical methods; (B) methods and details of the numerical temperature modelling; (C) methodological details of the derivation and statistical evaluation of permeability; (D) thermodynamic modelling details and extended figures; (E) details about the calculation of the redox mass balance in experiment; (F) an extended discussion of the H_2_ production from the heater and ingress into the capsule, including discussion of oxygen fugacities; and (G) additional supplementary figures. Further related datasets with raw and/or processed high-resolution data are available in repositories at 10.20350/DIGITALCSIC/16999 (Dataset 1: SEM–EDX-BSE-EBSD data), and 10.24416/UU01-WW5PS8 (Dataset 2: µ-CT data).
