# Supplementary material for: Controls of focused fluid release in subduction zones: insights from experimental dehydration of brucite vein networks in serpentinite
Source: Contrib Mineral Petrol. 2025 Apr 17;180(4):30. doi: 10.1007/s00410-025-02221-9 (PMC12006270; doi:10.1007/s00410-025-02221-9)
Supplement: Supplementary file 1 — Supplementary file1 (PDF 9267 KB) [file 410_2025_2221_MOESM1_ESM.pdf]

Supplementary material to: Controls of focused fluid release in subduction zones: insights from experimental dehydration of brucite vein networks in serpentinite

Manuel D. Menzel, Lisa Eberhard, Austin Arias, José Alberto Padrón-Navarta, Oliver Plümper

Contact: manuel.menzel@csic.es; l.eberhard@uu.nl

Content

A – Extended SEM and EBSD analytical methods

B – Numerical temperature model

C – Permeability derivation

D – Thermodynamic modelling

E – Redox mass balance in the experiment sample

F – Discussion of H_2 production / ingress and oxygen fugacities in the experiment

G – Additional supplementary figures

References

A – Extended SEM and EBSD analytical methods

Backscattered electron (BSE), energy dispersive x-ray spectroscopy (EDX) and electron back-scatter diffraction (EBSD) maps were obtained using a Zeiss EVO® MA 15 SEM equipped with an Oxford Instruments UltimMax-170 EDX and Nordlys Nano-EBSD detectors at the IACT–CSIC. For the SEM analysis, the starting material and post-experiment sample were diamond polished mechanically to 0.25 µm, followed by a final colloidal silica (50 nm) polishing on a Buehler Vibromet for 7 hours. No reaction between minerals in the samples and the colloidal silica was observed.

Simultaneous EBSD–EDX maps were acquired with a 20° incident angle between beam and sample surface, 17 kV acceleration voltage, 24 mm working distance and with step sizes of 1 – 3 µm on non-coated samples at low vacuum (10 Pa variable pressure) to avoid charging. EBSD maps of the starting material were acquired with a standard 4x4 binning to optimize

acquisition speed; whereas in the post-experiment sample EBSD maps were acquired with 2x2 binning for improved detection of Kikuchi patterns of brucite and olivine. Mean angular deviations (MAD) were in the range of 0.5 – 1.1° for brucite, 0.8 – 0.9° for antigorite, and 0.6 – 0.7° for olivine. Data treatment of EBSD results was performed using the Oxford HKL Channel5 software, including noise reduction (removal of wild spikes and successive filling of non-indexed pixels based on 8, 7 and 6 neighboring pixel orientations), and correction of phase misidentification based on the simultaneously acquired EDX data. Further correction of systematic misindexation of orientations due to pseudo-symmetry was performed for antigorite (60° and 120° [001]) and olivine (60° [100]), with a 5° threshold. In one detailed EBSD map, we divided the dataset into subsets corresponding to Mg-rich and Fe-rich olivine according to the EDX data. We used the Matlab-toolbox MTEX (version 5.11.2) (Bachmann et al., 2010) for grain modelling, plotting phase and orientation maps, calculating orientation distribution functions, and plotting pole figures. Grain reconstruction was done with a 10° segmentation angle, followed by the removal of small grains (≤ 8 pixel) as these are more prone to include measurement artefacts. For analysis of the crystallographic misorientations between neighboring brucite and olivine grains we performed an additional grain reconstruction step in a separate treatment so that phase boundary segments are well represented.

BSE and high-quality EDX mapping and point analysis were acquired on carbon-coated samples at high vacuum, 15 kV, and 8.5 mm working distance. EDX point analysis followed a standard-less approach, applying a beam current calibration on Co every 30 min, with measurement times of 35 s for silicates and 3-5 s for brucite and using high magnification (10.000x) to prevent errors from beam distortion. EDX mapping was obtained at 400x magnification with a step size of ~400 nm and 1 ms spectral acquisition time per pixel. To facilitate data processing, the EDX mapping data was binned (factor 2), resulting in a lower spatial resolution but improved spectral resolution. We used Fiji/ImageJ2 to obtain segmented phase by image processing of combinations of individual element and element ratio maps, and for particle analysis of brucite and magnetite grains/grain aggregates. To obtain a compositional map of X_{Mg} in olivine from the EDX count intensities of Mg and Fe, we applied composition-depended correction curves derived from the spot measurements in the mapped area.

Full EBSD data after correction of measurement artefacts and high-resolution BSE and EDX maps as well as a full-resolution version of the segmented phase map are contained in dataset 1, available at: http://doi.org/10.20350/DIGITALCSIC/16999.

B – Numerical temperature model

We calculated the temperature field in the piston-cylinder experiment numerically with the model initially published by Moarefvand et al. (2021), adjusted to the experimental setup used in this study (Fig. S1). The algorithm solves the heat equation in cylindrical coordinates:

$$\rho\, C_p(T)\,\frac{\partial T}{\partial t} = \frac{1}{r}\frac{\partial}{\partial r}\left(k(T)\; r\;\frac{\partial T}{\partial r}\right) + \frac{\partial}{\partial y}\left(k(T)\,\frac{\partial T}{\partial y}\right) + Q. \qquad \text{(R-S1)}$$

Here, ρ is the density at the reference temperature of 20 °C, $C_p(T)$ the heat capacity at temperature T, $k(T)$ the thermal conductivity at temperature T, and r and y the cylindrical coordinates. Q is the heat production term. We used temperature-dependent heat capacities and thermal conductivities. Densities were held constant, i.e., no compressibility and thermal expansion was included. Preliminary calculations showed that using pressure- and temperature-depend densities has negligible effects on the final temperature field. Table S1 summarizes all material parameters used in the calculation.

The cylindrical geometry of the setup allowed us to calculate a half-model (2001 x 2001 nodes), which speeds up the calculation. The initial temperature was set to 25 °C, corresponding to the temperature of the cooling water. Boundary conditions were set to constant temperature (25°C) at the outer boundaries and zero heat flux at the inner boundary. Heat production was restricted to the graphite heater. Time steps were set to 10 seconds and the model was run until the temperature at the thermocouple junction converged to the experimental temperature and no more temperature changes were observed. Fig. S2 shows the final temperature field.

Table S1: *Physical properties of materials used in the numerical simulation. References are as followed: 1 Arblaster (2016), 2 Powell et al. (1966); 3 Holland and Powell (2011); 4 Gummow and Sigalas (1988); 5 Ghosh et al. (2015); 6 Hernlund et al. (2006); 7 Osako et al. (2010); 8 Kanamori et al. (1968); 9 Grizinvold et al. (1988); 10 Williams (1998); 11 Wattanasarn and Seetawan (2013); 12 Slifka et al. (1998).*

material	density [kg / m^3]	heat capacity [J / (kg K)]		thermal conductivity [W / (m K)]		ref.
	ρ	C_p0	$C_p\,(T)$	$k0$	$k\,(T)$	
steel	8000	469.32	-0.9e^{-4} T^2 + 3.075e^{-1} T + 386.95	21.9	2.16e-^{2} T + 15.5	1, 2
Talc	2778	841.2	2.03e^{-12} T^5 - 9.66e^{-9} T^4 + 1.79e^{-5} T^3 - 1.66e^{-2} T^2 + 8.07 T - 506	1.67	1.405 + 0.001 T	3, 4
Borosilicate	2230	846.5	1.5894 T + 380.82	0.91	0.495 + 0.0014 T	5

graphite	2200	685	$-1.49e^{-10}\ T^4 + 1.16e^{-6}\ T^3 - 3.39e^{-3}\ T^2 + 4.58\ T - 394$	109.84	$-6.54e^{-9}\ T^3 + 5.43e^{-5}\ T^2 - 1.49e^{-1}\ T + 149$	3, 6
serpentine	2600	958.9	$2.58e^{-6}\ T^3 - 5.81e^{-3}\ T^2 + 4.83\ T - 22.4$	2.61	$2.72 - 3.78e^{-4}\ T$	7
alumina	3950	759.6	$330 \log(T) - 1.07e^{3}$	37.19	$4.10e^{-11}\ T^4 - 1.90e^{-7}\ T^3 + 3.33e^{-4}\ T^2 - 2.69e^{-1}\ T + 91.9$	3, 8
gold	21400	129	$130.79 - 8.7e^{-3}\ T + 2.51e^{-5}\ T^2 - 1.91e^{5}\ T^{-2}$	320	$-2.7e^{-8}\ T^3 + 1.2e^{-5}\ T^2 - 3.5e^{-2}\ T + 330$	3, 1
W-carbide	15630	165.98	$51.2 \log(T) - 126$	57.7	$-1.38e^{-2}\ T + 61.7$	9, 10
MgO	3550	914.38	$1.182e^{3} + 0.142\ T - 2.182e^{-5}\ T^2 + 2.6075e^{-9}\ T^3 - 2.6e^{7}\ T^{-2}$	43.22	$86.867 - 0.192\ T + 1.608e^{-4}\ T^2 - 4.656e^{-8}\ T^3$	11, 12

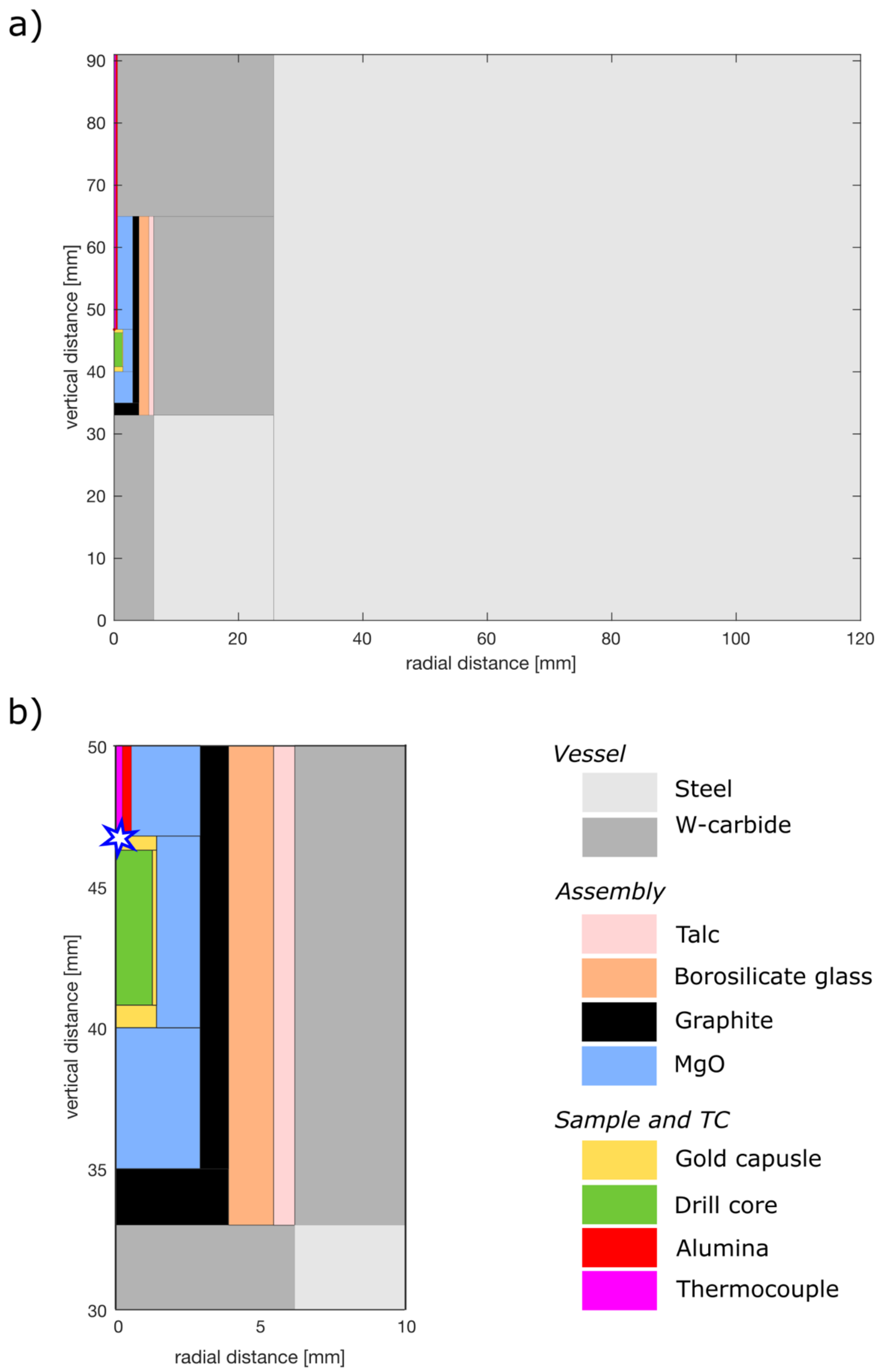

Figure S1: *Numerical model setup.* ***a)*** *Full experimental setup including the pressure vessel. Temperature at the top, bottom and right side was fixed to 25 °C.* ***b)*** *Zoom in to the experimental assembly. Modelled temperature at the thermocouple junction (white star on top of the capsule) is compared to the experimental temperature.*

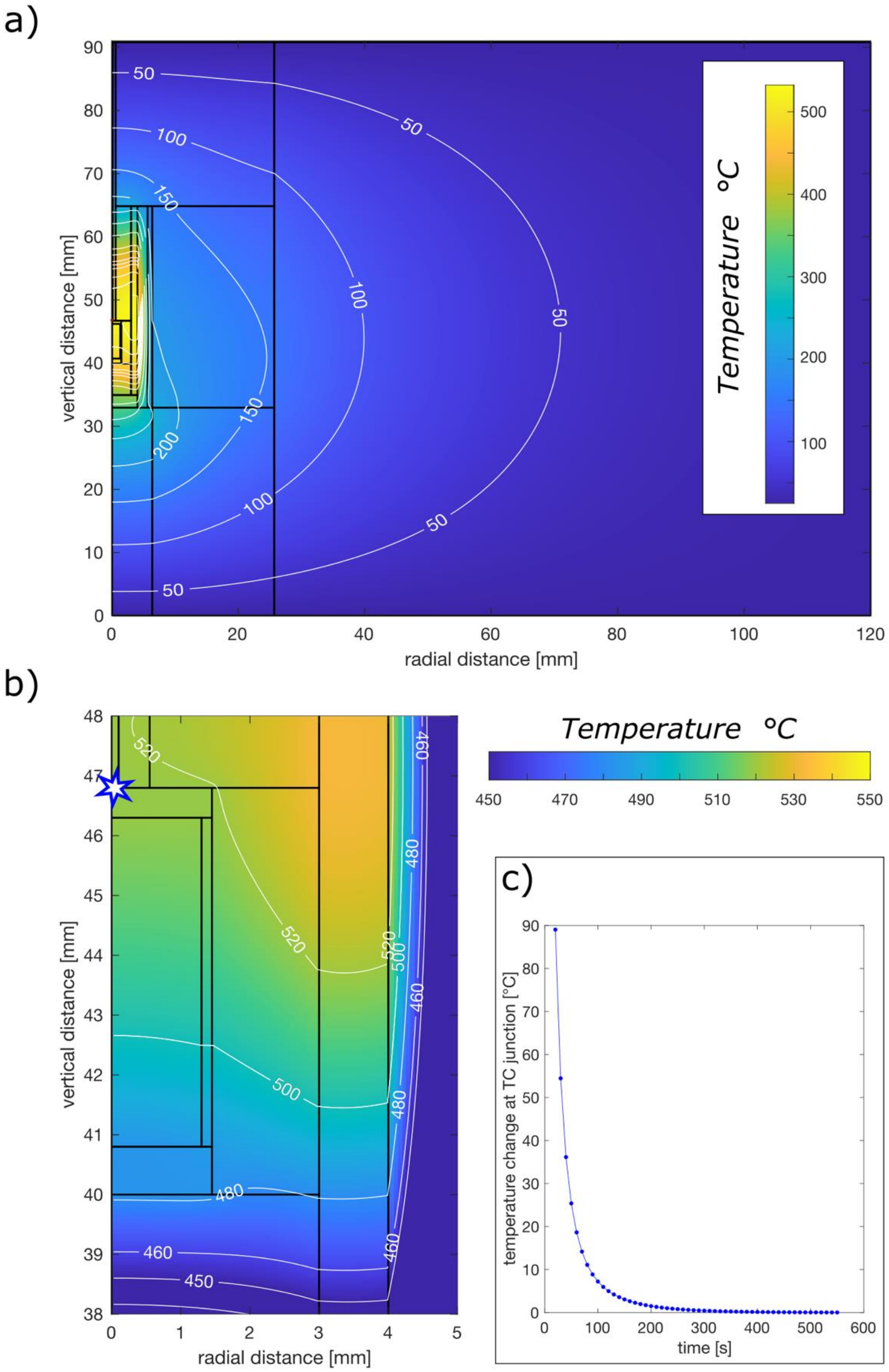

Figure S2: *Calculated temperature field.* ***a)*** *Temperature in the vessel shows a steep gradient around the heater.* ***b)*** *The hot spot is close to the sample top. White star indicates position of the thermocouple junction. The sample temperature gradient is ~40 °C from top to bottom.* ***c)*** *Temperature change at the thermocouple junction for each time step with respect to the previous time step. Convergence is reached after 400 s (=40 steps).*

C – Permeability derivation

The partial dehydration in our experiment formed a monomineralic olivine-pore aggregate around former brucite grains, along the brucite vein rims, and around magnetite (see main text for examples). We calculated the permeability of this olivine-pore aggregate in the most reacted region of our sample (a 718 x 883 x 1863 μm^3 sub-volume from the top of the reacted sample) by randomly sampling domains for which the statistical properties of the assemblage converge and the effective physical properties of the heterogeneous medium can be described (Torquato 2002; Kanit et al., 2003).

Fig. S3 shows how the domains were sampled from the 3D µ-CT scans in four steps: 1) We divided the segmented 3D µ-CT scans into two assemblages, whereby one assemblage is composed of the olivine-pore aggregate and the other assemblage includes all other labeled phases in the bulk sample (antigorite–brucite, magnetite, spinel). 2) We extracted the image skeleton of the olivine-pore aggregate using the image processing software FIJI (Schindelin et al., 2012), which defines the center of the regions filled with this aggregate. 3) Random sampled domains with variable box sizes were taken around the image skeleton. If, within a sampled box, the volume fraction of the olivine-pore aggregate was greater than 90%, that box was kept as a valid sample domain. Otherwise, the domain was rejected. The box sizes used for this initial sampling range from 20–180 voxels in length (27 - 243 µm), and we took 100 samples for each box size. 4) We then trimmed all the sampled boxes by 20 % to avoid accidental sampling of a non-porous wall, which can cause unexpected behavior while calculating the permeability. The effective box sizes are thus ranging from 16–144 voxels in length (21.6 - 194.4 µm).

We note here that step 3) has a high risk of sampling non-unique domains for large box sizes, as it becomes increasingly more difficult to find 100 unique domains with increasing box sizes. Fig. S4 and S5 highlight the tendency of increasing box sizes to repeatedly sample the same domain. With a box size of 20 voxels in length, almost all sampled domains are unique, whereas with a box size of 80 voxels in length, less than 50 % of the sampled domains are unique and with a box size of 180 voxels per length, all sampled domains overlap to a large extend.

With step 4) we aim to reduce accidental sampling of non-porous material surrounding the olivine-pore aggregate. Fig. S6 illustrates the effect of this step by showing the probability density of the volume fraction of the olivine-pore aggregate in trimmed sample domains. For smaller box sizes it is likely to find samples composed only of olivine-pore aggregate. Larger

boxes, however, are approaching the 90 % sampling threshold even after the trimming step. Consequently, box sizes ≥144 voxels approach the maximum width of the olivine-pore aggregate surrounding the brucite vein, which can be up to 200 µm wide according to µ-CT and SEM analyzes (Fig. 3; Fig. 4a; Fig. 7 in main text).

For all sampled domains we calculated the porosity ϕ and the two-point correlation function curves, $S_2(r)$ (Torquato 2002; Jiao et al., 2007) on the trimmed volumes. ϕ is defined as the volume fraction of pore within the sampled olivine-pore aggregates. $S_2(r)$ is defined as

$$S_2^j(r) = \mathbb{P}(x \in V^j, x + r \in V^j), \tag{R-S2}$$

where $\mathbb{P}$ indicates a probability, j describes the phase of interest, r is the displacement from position x, and V^j describes a set of all positions where the phase is j (Yeong & Torquato, 1998). $S_2(r)$ can be effective at identifying representative volume elements of porous materials as the function bounds are characterized by $S_2(0) = \phi$ and $\lim_{r\to\infty} S_2(r) = \phi^2$. We therefore expect a sufficiently large and representative sample to produce curves that approach ϕ^2. Fig. S7 shows the two-point correlation with the average porosity of 0.304 in the limit of $r \to 0$ and the convergence to ϕ^2 with increasing r. Overall, the two-point correlation function for each cartesian axis (reference frame as indicated in Fig. S3) are similar. However, there is a slight difference in the two-point correlation function along the X axis, suggesting slight structural anisotropy perpendicular to the brucite vein. In Fig. S8 we show the final porosity for the sampled domains. We find that smaller box sizes reveal larger variations in porosity, which can be explained by the fact that box sizes of 16 voxels (21 µm) are likely to sample only a few grains (olivine grain sizes: 5 - 20 µm). With increasing box sizes, the porosity converges towards an average porosity of 0.304, which is consistent with an estimated porosity of 0.30 - 0.36 from thermodynamic calculations (Fig. 8 - 9 in main text, in the limit of $X_{Brc} \approx 0.23$, being representative for an ideal Atg-Brc interface).

The permeability for the sampled domains was found by solving the equations for Stokes' flow,

$$\mu \nabla^2 \bar{u} - \nabla P + \bar{b} = 0 \tag{R-S3}$$
$$\nabla \cdot \bar{u} = 0, \tag{R-S4}$$

through the porous medium in the trimmed boxes three times. Here, μ is the dynamic viscosity of the fluid, $\bar{u}$ is the velocity, P is pressure, and $\bar{b}$ is a body force acting on the fluid. Each time a unitary body force is assigned parallel to either the X, Y, or Z axes. Then, the system is homogenized using the average flow velocities and Darcy's law,

$$\langle \bar{u} \rangle = -\frac{1}{\mu} \bar{K} \langle \nabla P - \bar{\bar{b}} \rangle, \quad \text{(R-S5)}$$

where, $\langle \bar{u} \rangle$ is the average velocity within the target domain, $\langle \nabla P - \bar{\bar{b}} \rangle$ are the assigned boundary conditions, and $\bar{K}$ is the permeability tensor. We applied the GPU-accelerated finite element solver of Lopes et al. (2023) to solve for the full permeability tensor of all sample boxes using periodic boundary conditions. For each sample we applied 4x voxel refinement to reduce solid-pore interface effects when solving for the velocities. We then diagonalized the permeability tensors by finding the eigenvalues and eigenvectors to determine the principal permeabilities. Fig. S9 shows the final permeabilities along the principal axes for variable box sizes, which converge in the range of 5 x 10^{-14} to 1 x 10^{-13} m^2.

In Fig. S10, finally, we compare the principal directions of the permeability tensor (*v1*, *v2*, *v3*) for the variable box sizes with the cartesian coordinates of the sample. From the above discussions, we found that small box sizes perform better in terms of their uniqueness (Fig. S4 - S5), but suffer from large uncertainties in calculated properties due to grain size-effects (Fig. S6 - S9). For larger box sizes the physical properties converge, resulting in an apparent higher accuracy, but they are prone to non-uniqueness (Fig. S4 - S5) and are possibly influenced by non-porous material (Fig. S6 - S9). We thus infer that the permeability values for the olivine-pore aggregate from intermediate box sizes of 48 to 112 px in length are most robust. Here, the permeability is slightly anisotropic with the lowest permeability (K1) approximately parallel to the normal of the brucite vein (X axis), whereas v2 and v3 have similar magnitude (K2 and K3) and lie within the Y-Z plane (parallel to the brucite vein). We interpret these findings as a sign of high nucleation density at the vein interface (c.f. Fig. 4e and Fig. 6 in main text), which creates a dense layer and possibly reduces the permeability perpendicular to vein, while fluid flow parallel to the vein is promoted. Local orientated growth of olivine perpendicular to the vein (c.f. main text for detailed discussion) could also affect the permeability and cause the structural anisotropy observed in the two-point correlation functions. However, the differences are small and the overall permeability in the olivine-pore aggregate may overall be approximated as isotropic.

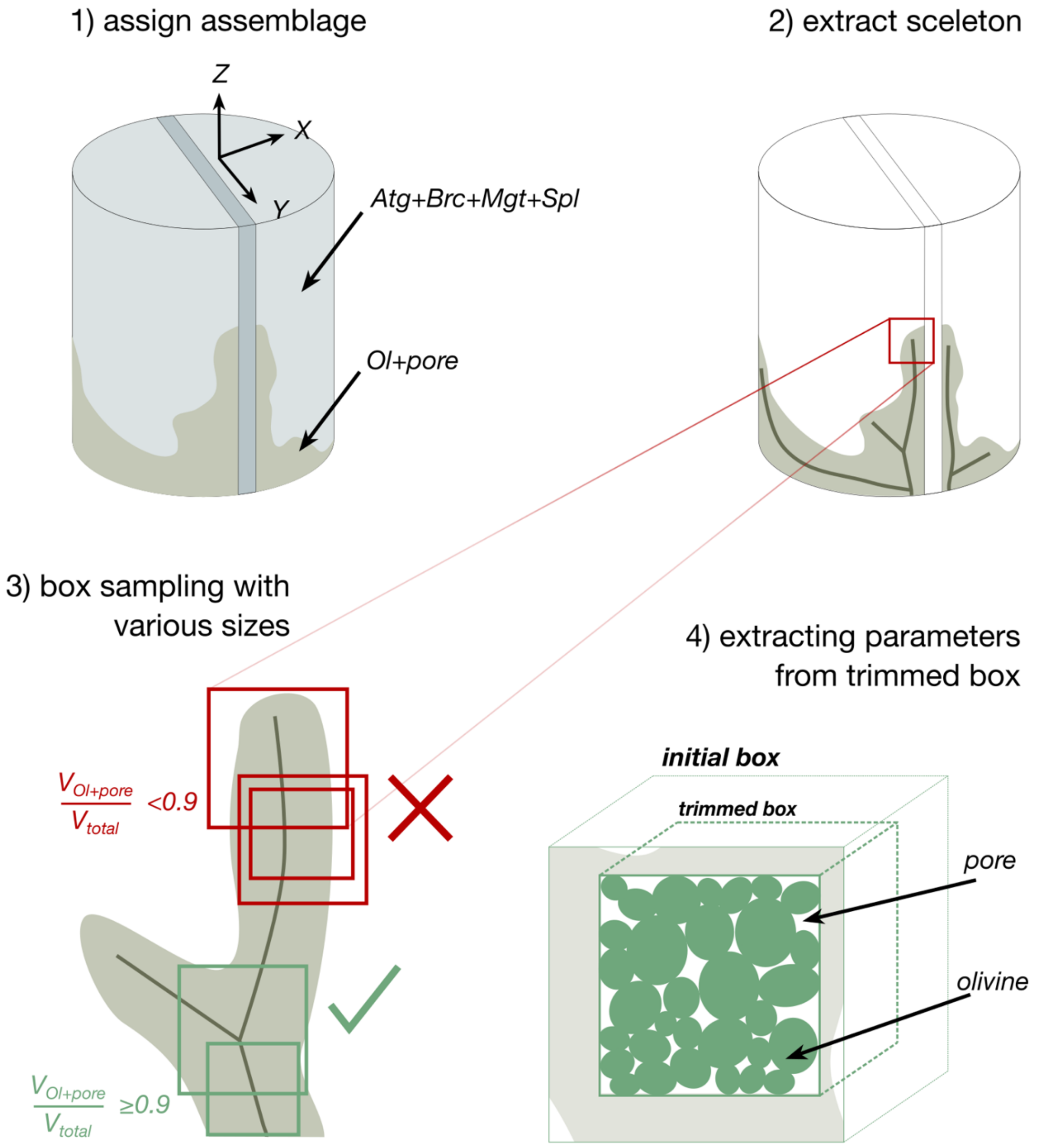

Figure S3: *Flow chart showing the steps to sample random domains from 3D µ-CT scans for further porosity and permeability calculation. 1) The segmented 3D µ-CT scans are divided into two assemblages. 2) The image skeleton of the target assemblage is extracted. 3) Box sampling across the skeleton. Only boxes containing more 90 % of the target assemblage are considered. 4) The boxes are trimmed and physical parameters extracted.*

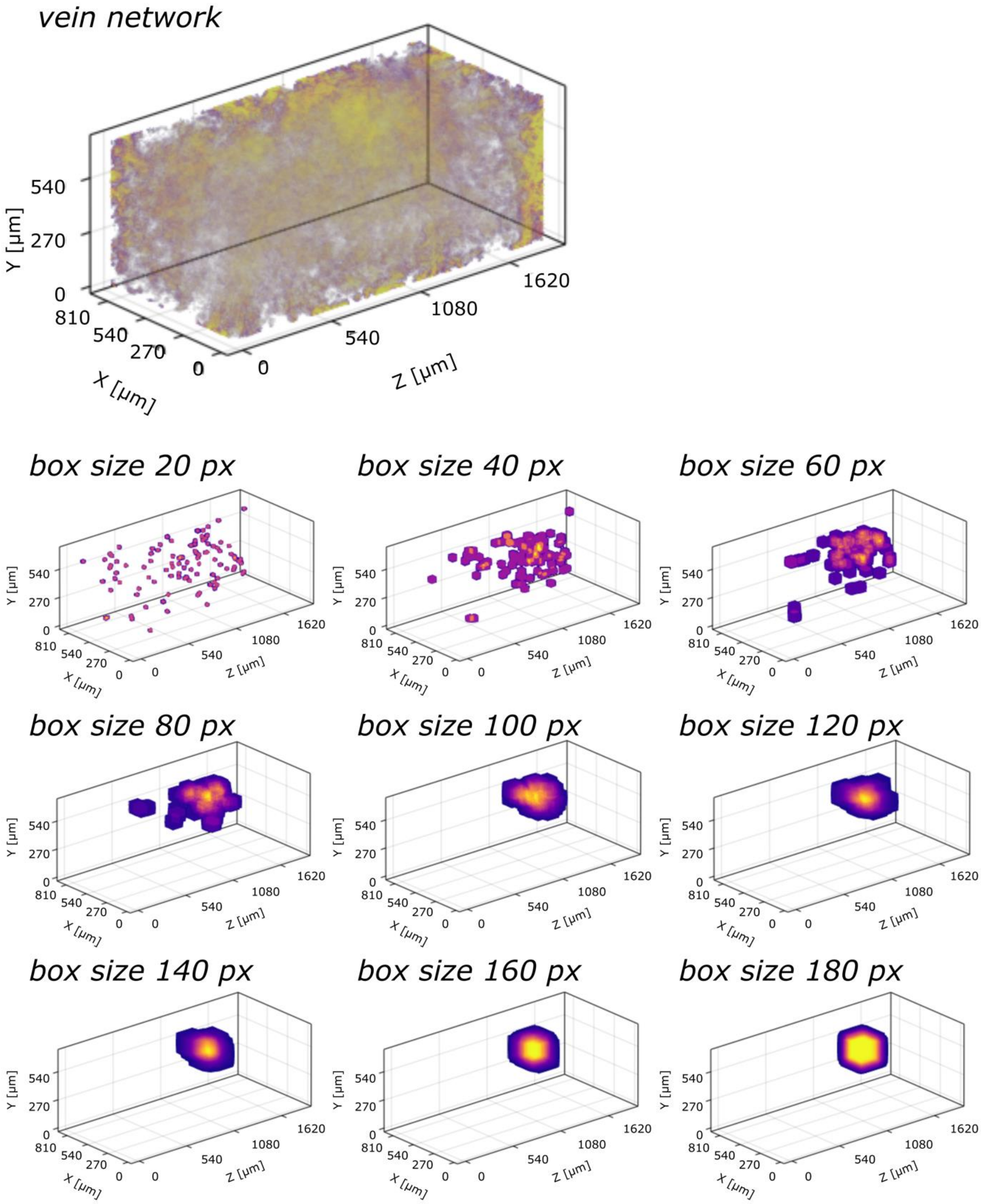

Figure S4: *3D rendering of the vein network and sampled domains for each box size. In the renderings of the sampled domains, yellow regions indicate overlap during the sampling. Small box sizes are almost always unique and show no overlap, whereas larger box sizes tend to be non-unique and show large overlap.*

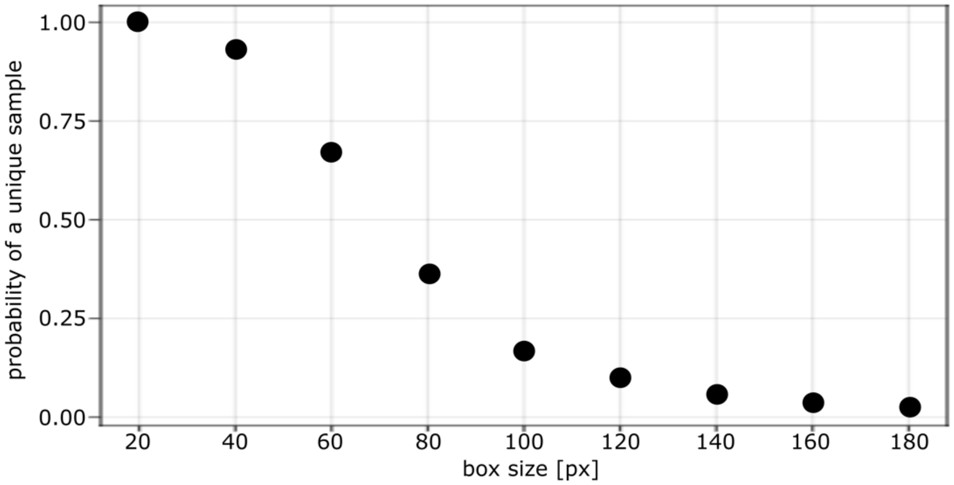

Figure S5: *Probability for each box size to sample a unique domain. The probability is defined as the quotient of the number of unique voxels in all sampled boxes for a set box size and the total volume of voxels sampled. In the case of the smallest boxes, almost all samples will be unique. With increasing box size, the total available unique sampling volume decreases to zero as it reaches the natural limit of the bulk sample.*

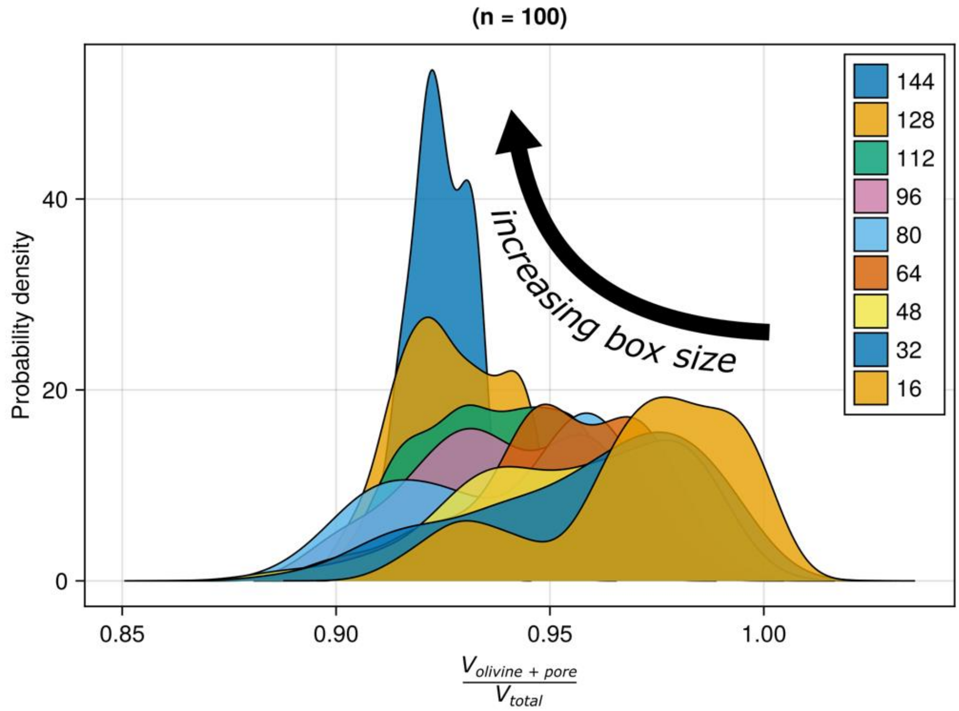

Figure S6: *Probability density of the volume fraction of olivine-pore aggregate within trimmed sample domains with different box sizes. Smaller box sizes are more likely to contain only space defined as olivine or pore, whereas the largest box sizes are approaching the 90% sampling threshold. This suggests box lengths greater than 144 voxels are approaching the maximum width of the olivine-pore aggregate in the bulk sample.*

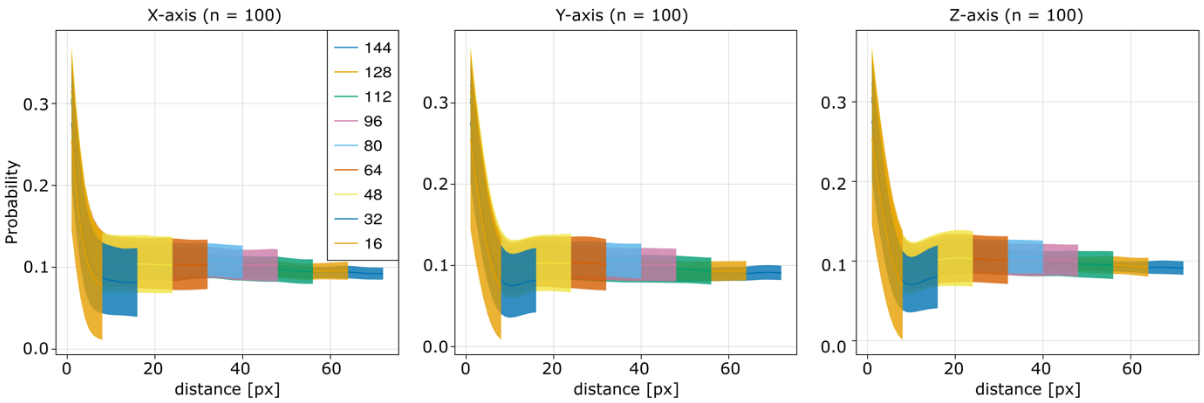

Figure S7: *The $S_2(r)$ curves for X, Y, and Z axes of sampled domains (trimmed boxes). The lines indicate the average curve for each box size surrounded by a ribbon representing the standard deviation. For box sizes ≥48 voxels, the curves converge to ϕ^2. The curves calculated across the X axis differ slightly to those in Y and Z, suggesting slight structural anisotropy.*

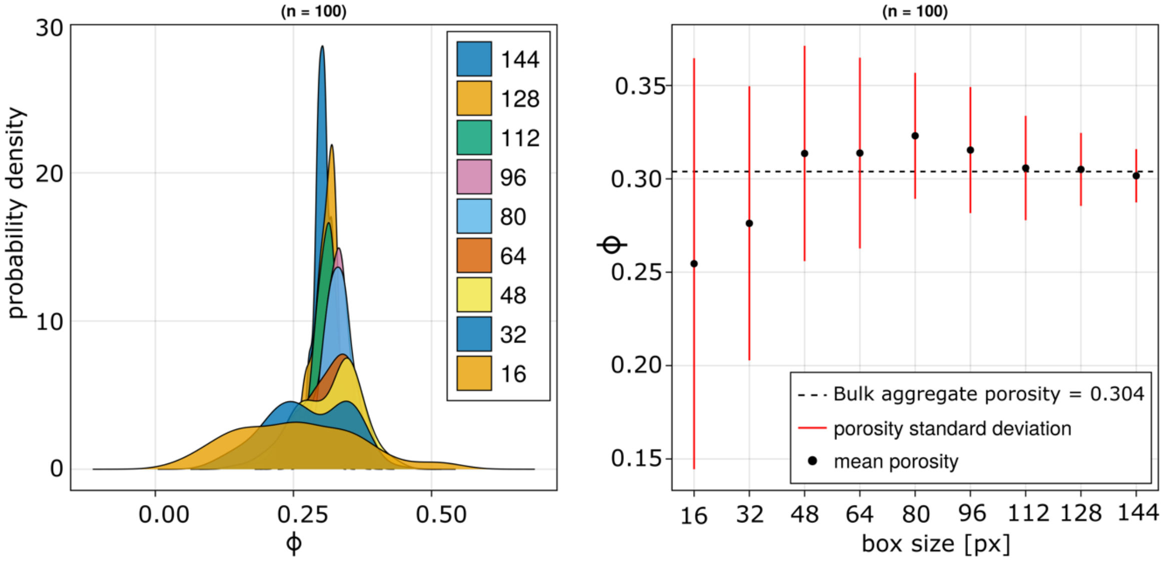

Figure S8: *(Left) Probability densities of the porosity for the sampled domains taken for each box size (trimmed boxes). The smaller box sizes exhibit the greatest variation, and the porosity converges with increasing box size. (Right) Average and standard deviation of the measured porosity for the sampled domain with the X- axis indicating the box size (trimmed box). The largest box sizes converge towards the bulk average porosity (0.304).*

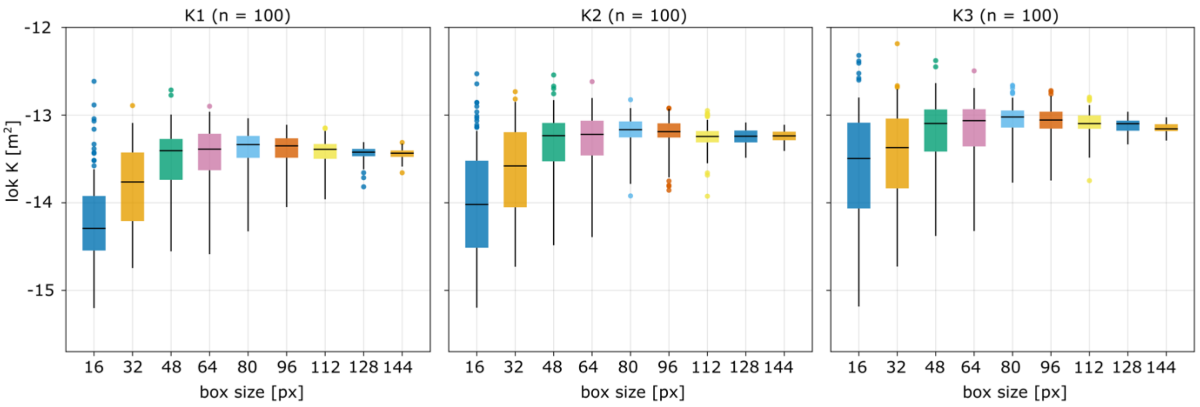

Figure S9: *Box plots of the calculated principal permeability for the sampled domains (trimmed boxes). Each of the principal permeability values converges at larger box sizes to similar values between 5 × 10−14 to 1 × 10−13, suggesting the effective permeability of the olivine aggregate is isotropic.*

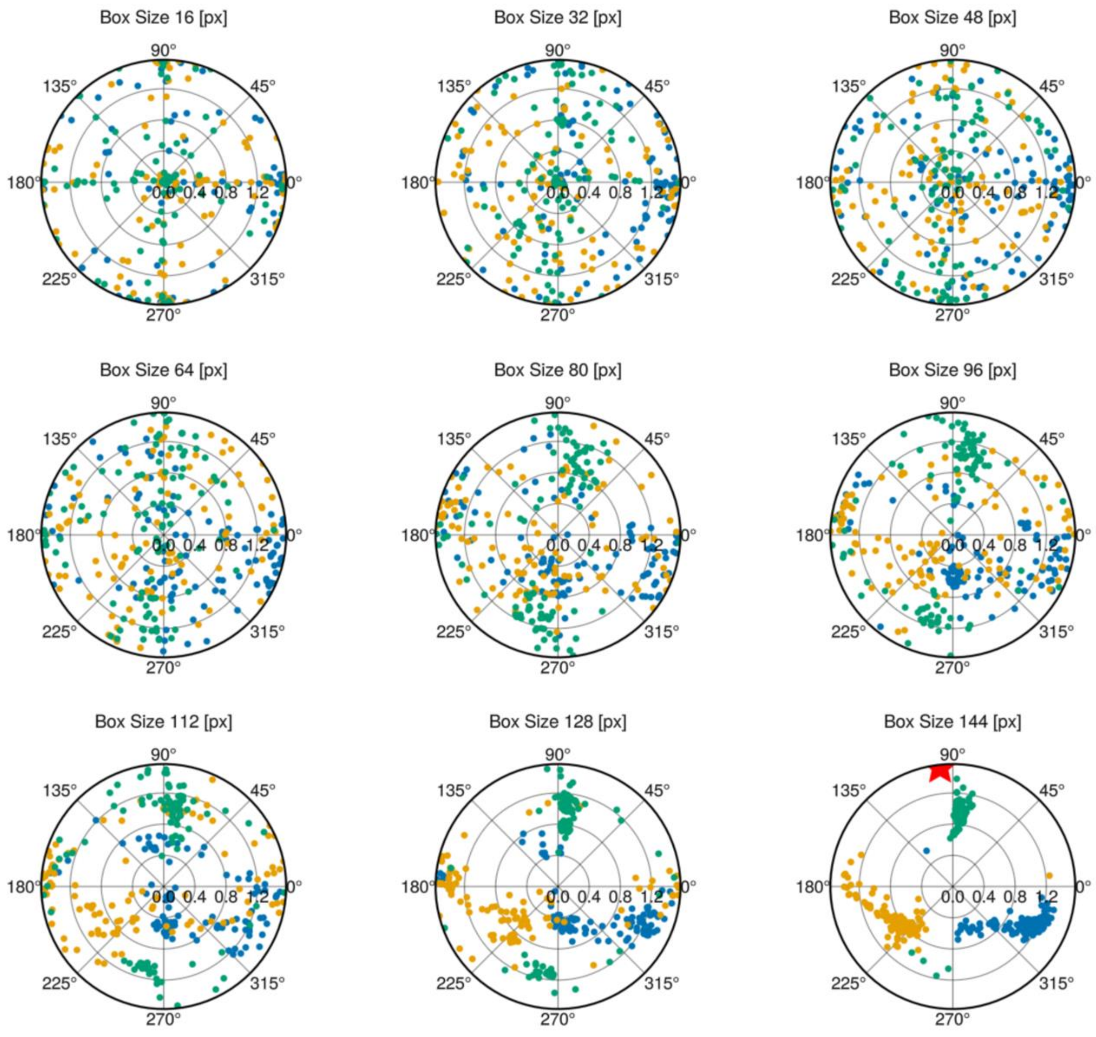

Figure S10*: Principal directions of the calculated permeability tensors for each box size (trimmed boxes). On each pole figure the azimuth angle θ = 0◦ and altitude value φ = π/2 indicates a the Z axis, θ = 90◦ and φ = π/2*

is the X axis, and a vector of any azimuth with φ = 0 is aligned to the Y axis. v1 is green, v2 is orange, and v3 is blue. The pole figure of box size 144 pixels contains a red star representing the normal vector of the brucite vein.

D – Thermodynamic modelling

Table S2. *Bulk rock major element composition of the starting material (sample ADV-15) as reported in Menzel et al. (2018), and the derived simplified compositions in the FMASH-O chemical system used for thermodynamic modelling. Model composition 2 (X_{Brc} = 0) was derived by extrapolating the compositional vector between compositions 1 and 3 towards a brucite-free composition, so that the endmember compositions 2 and 3 are similar to the local bulk compositions of the brucite-free bastite domain (X_{Bcr} = 0) and of serpentine-free brucite vein (X_{Brc} = 1) shown in Fig. 8a, main text.*

	**Atg-serpentinite Adv 15** [a]	**Model comp. 1 Adv 15 simplified**		**Model comp. 1* Adv 15 simplified**	**Model comp. 2* Serpentinite (X_{Brc}=0)**	**Model comp. 3* Brc vein (X_{Brc}=1)**
**SiO_2 (wt%)**	39.42	39.42	**Si**	6.5636	7.2626	0.0000
**Al_2O_3**	0.38	0.38	**Al**	0.0746	0.0825	0.0000
**Fe_2O_3**	4.54	4.54	**Fe**	0.7768	0.8595	0.7952
**FeO**	1.49	1.49	**Mg**	10.1103	9.5381	15.9302
**MnO**	0.09		**H_2** [e]	7.4409	6.3069	16.4440
**MgO**	40.73	40.73	**O_2** [f]	15.9259	15.8336	16.7298
**CaO**	0.07					
**K_2O**	0.01			(Fig. 9)	(Fig. 8)	(Fig. 8)
**S**	0.02					
**CO_2**	0.22					
**H_2O**	12.46 [b]	13.4 [d]				
**Cr (ppm)**	2737					
**Ni (ppm)**	2477					
**Total**	100.07	99.96				
**Fe^{3+}/Fe_{total}** [c]	0.73	0.73				

[a] Measured whole rock composition (see Menzel et al., 2018).

[b] estimated from LOI (loss on ignition after correction for oxidation gain) minus CO_2

[c] Calculated molar ratio

[d] including excess H_2O to assure fluid saturation at all relevant P-T conditions

[e] shown here for X_{H2} = 0

[f] Oxygen based on redox budget with Fe^{3+}/Fe_{total} = 0.73 for X_{H2} = 0

* molar elemental composition normalized to 1 kg rock

Table S3. Perple_X input used for calculation of Fig. 8. *The thermodynamic data file is a slightly modified version of DEW19HP622ver_elements.dat with the appropriate make definitions for antigorite solution models activated and the adjusted dqf parameter of the f3clin endmember of chlorite, as described in the methods section of the paper. The perplex_option_lag.dat is the standard perplex option file but with aqueous lagged speciation activated. The input composition below is for* $X_{H2} = 0$*; for higher* X_{H2} *the respective molar quantity was added to* H_2 *in both columns.*

```
DEW19HP622ver_elements_LE.dat     thermodynamic data file
print     | no_print suppresses print output
plot      | obsolete 6.8.4+
solution_model_mod.dat     | solution model file, blank = none
T-X C0 = model-comp2 (Serp+Mag+-tlc) C1= Brc+Mag, Fe3/tot 0.73, 1.5GPa.
perplex_option_lag.dat     | Perple_X option file
    5 calculation type: 0- composition, 1- Schreinemakers, 2 - liquidus/solidus, 3- Mixed, 5- gridded
min, 7- 1d fract, 8- gwash, 9- 2d fract, 10- 7 w/file input, 11- 9 w/file input, 12- 0d infiltration
    0 unused place holder, post 06
    0 unused place holder, post 06
    0 unused place holder, post 06
    0 unused place holder, post 06
    0 unused place holder, post 06
    0 unused place holder, post 06
    0 unused place holder, post 06
    0 unused place holder, post 06
    0 unused place holder, post 06
    0 number component transformations
   21 number of components in the data base
    0 component amounts, 0 - mole, 1 mass
    0 unused place holder, post 06
    0 unused place holder, post 06
    0 unused place holder, post 05
    0 ifug EoS for saturated phase
    2 gridded minimization dimension (1 or 2)
    0 special dependencies: 0 - P and T independent, 1 - P(T), 2 - T(P)
 0.00000      0.00000      0.00000      0.00000      0.00000     Geothermal gradient polynomial coeffs.

begin thermodynamic component list
Si    2  7.26260      0.00000      0.00000     molar amount
Al    2  0.08250      0.00000      0.00000     molar amount
Fe    2  0.85950     0.795200      0.00000     molar amount
Mg    2  9.53810      15.9302      0.00000     molar amount
H2    2  6.30690     16.44400      0.00000     molar amount
O2    2  15.8336      16.7298      0.00000     molar amount
end thermodynamic component list

begin saturated component list
end saturated component list

begin saturated phase component list
end saturated phase component list

begin independent potential/fugacity/activity list
end independent potential list

begin excluded phase list
h2oL
H2,aq
O2
HFeO2-
mft
end excluded phase list


begin solution phase list
O(JH)
Cpx(JH)
Opx(JH)
Atg(LE)
Chl(W)
B
T
COH-Fluid
end solution phase list

   15000.0        793.000        0.00000        0.00000        0.00000     max p, t, xco2, mu_1, mu_2
   15000.0        743.000        0.00000        0.00000        0.00000     min p, t, xco2, mu_1, mu_2
   0.00000        0.00000        0.00000        0.00000        0.00000     unused place holder post 06

 2  1  4  5  3   indices of 1st & 2nd independent & sectioning variables
```

Table S4. Perple_X input used for calculation of Fig. 9.

```
DEW19HP622ver_elements_LE.dat     thermodynamic data file
print     | no_print suppresses print output
plot      | obsolete 6.8.4+
solution_model_mod.dat     | solution model file, blank = none
Adv15 no Cr,Ca,C. C0=measured Fe3/tot 0.73 C1= + 0.5 mol H2, lagged, 0.3wt% H2O added. brc-out at 1.5GPa
perplex_option_lag.dat     | Perple_X option file
    5 calculation type: 0- composition, 1- Schreinemakers, 2 - liquidus/solidus, 3- Mixed, 5- gridded
min, 7- 1d fract, 8- gwash, 9- 2d fract, 10- 7 w/file input, 11- 9 w/file input, 12- 0d infiltration
    0 unused place holder, post 06
    0 unused place holder, post 06
    0 unused place holder, post 06
    0 unused place holder, post 06
    0 unused place holder, post 06
    0 unused place holder, post 06
    0 unused place holder, post 06
    0 unused place holder, post 06
    0 unused place holder, post 06
    0 number component transformations
   21 number of components in the data base
    0 component amounts, 0 - mole, 1 mass
    0 unused place holder, post 06
    0 unused place holder, post 06
    0 unused place holder, post 05
    0 ifug EoS for saturated phase
    2 gridded minimization dimension (1 or 2)
    0 special dependencies: 0 - P and T independent, 1 - P(T), 2 - T(P)
 0.00000      0.00000      0.00000      0.00000      0.00000     Geothermal gradient polynomial coeffs.

begin thermodynamic component list
Si    2  6.56360      6.56360      0.00000     molar amount
Al    2 0.746000E-01 0.746000E-01  0.00000     molar amount
Fe    2 0.776800     0.776800      0.00000     molar amount
Mg    2  10.1103      10.1103      0.00000     molar amount
H2    2  7.44090      7.94090      0.00000     molar amount
O2    2  15.92589     15.92589     0.00000     molar amount
end thermodynamic component list


begin saturated component list
end saturated component list


begin saturated phase component list
end saturated phase component list


begin independent potential/fugacity/activity list
end independent potential list


begin excluded phase list
h2oL
H2,aq
O2
mft
end excluded phase list


begin solution phase list
O(JH)
Cpx(JH)
Opx(JH)
Atg(LE)
Chl(W)
B
T
COH-Fluid
end solution phase list

   15000.0        793.000        0.00000        0.00000        0.00000      max p, t, xco2, mu_1, mu_2
   15000.0        743.000        0.00000        0.00000        0.00000      min p, t, xco2, mu_1, mu_2
   0.00000        0.00000        0.00000        0.00000        0.00000      unused place holder post 06

 2  1  4  5  3   indices of 1st & 2nd independent & sectioning variables
```

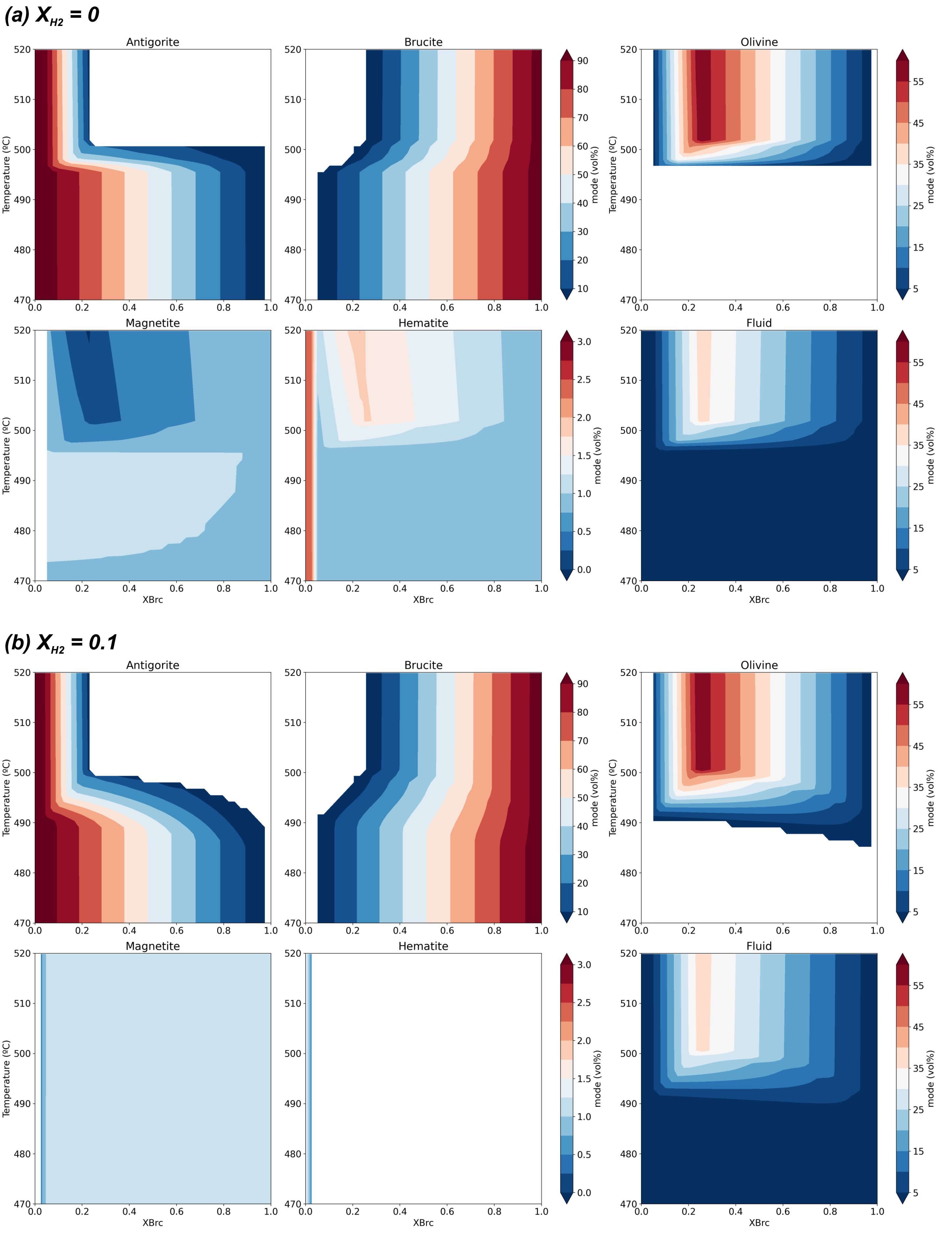

Supplementary Fig. S11. Predicted phase proportions (vol%) as a function of the local effective equilibration volume‘s composition from serpentinite to brucite-vein (X_{Brc}) at different X_{H2} (c.f. Fig. 8).

(c) X_{H2} = 0.2

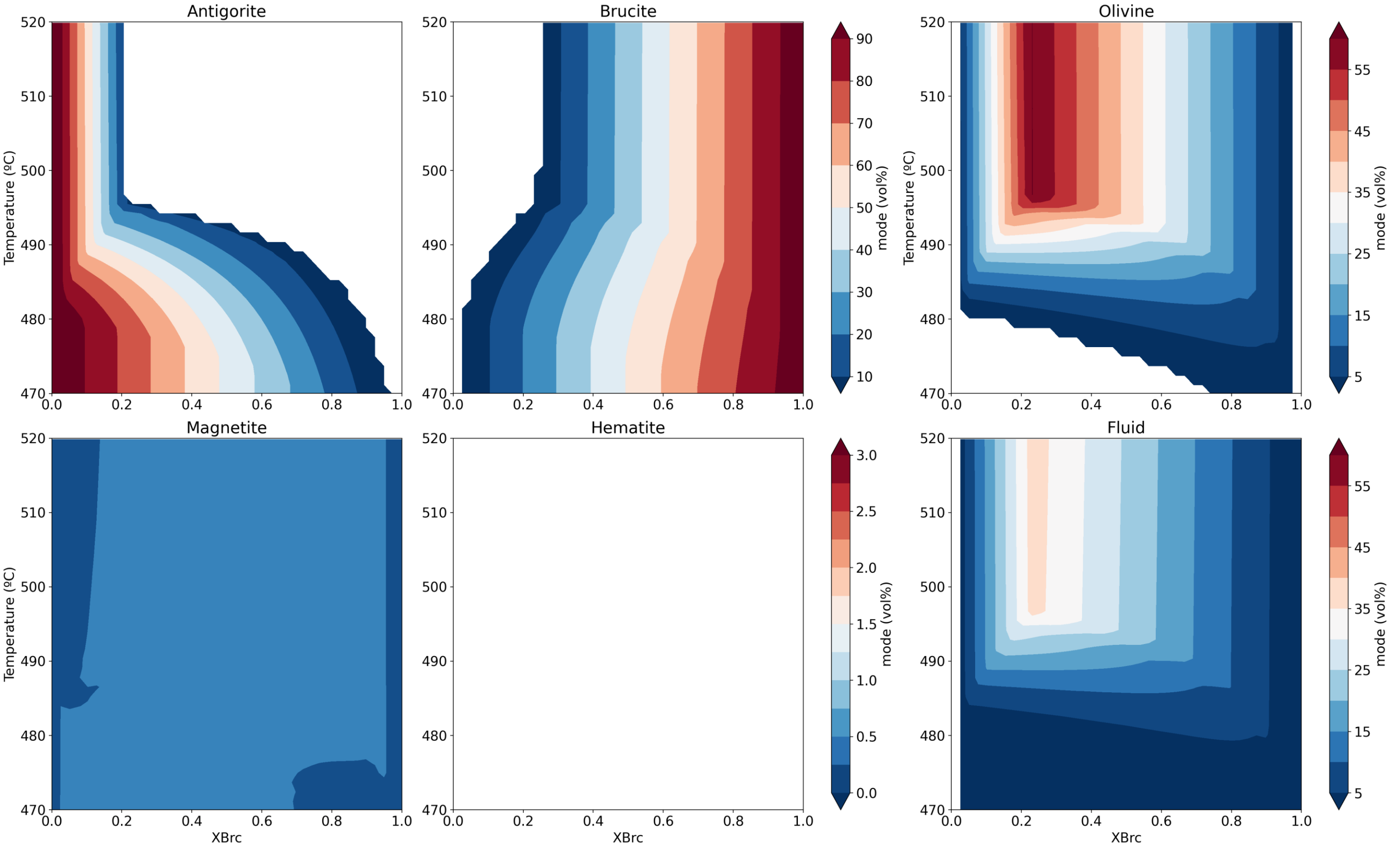

(d) X_{H2} = 0.3

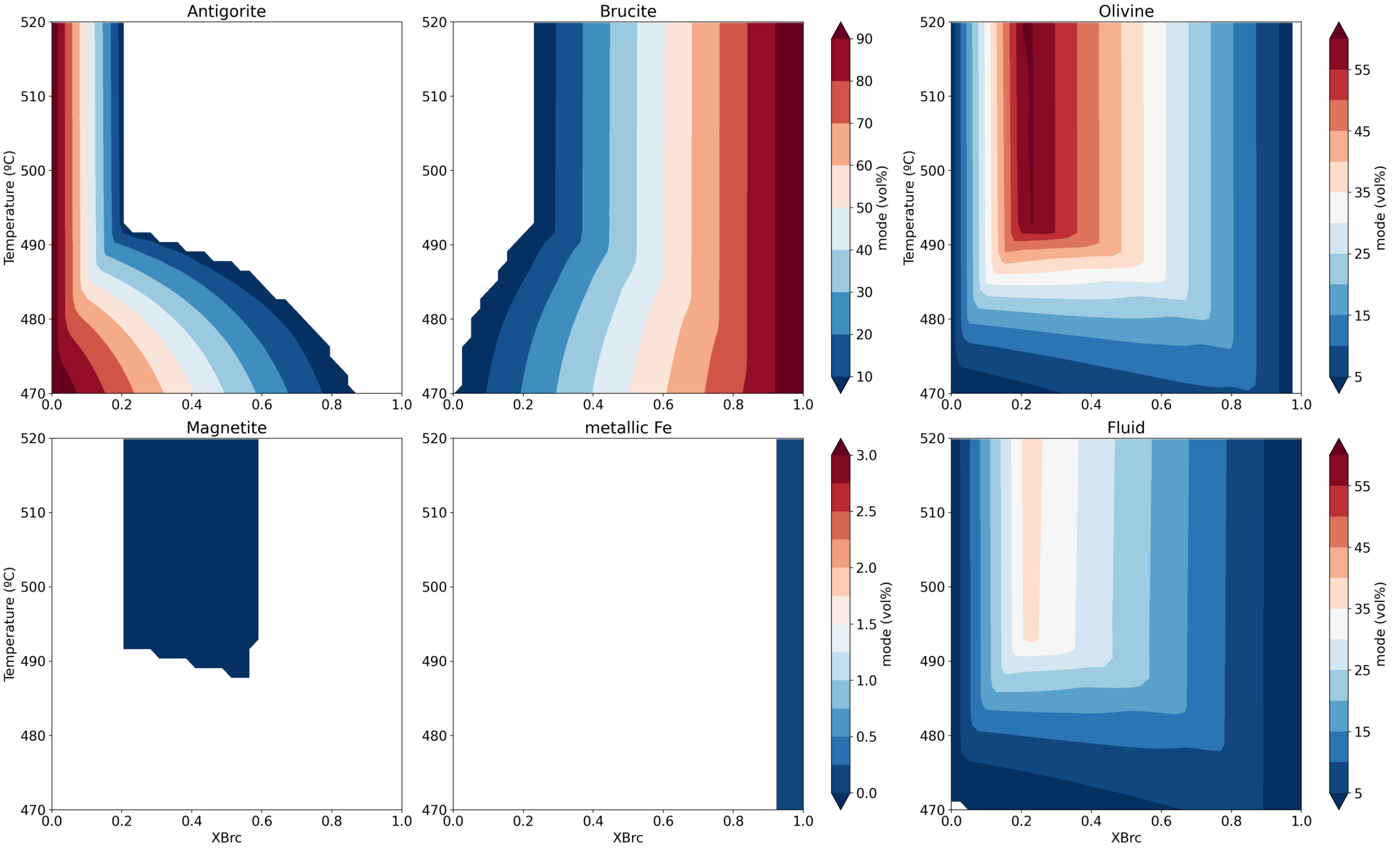

Supplementary Fig. S11 (continued). Predicted phase proportions (vol%) as a function of the local effective equilibration volume‘s composition from serpentinite to brucite-vein (X_{Brc}) at different X_{H2} (c.f. Fig. 8).

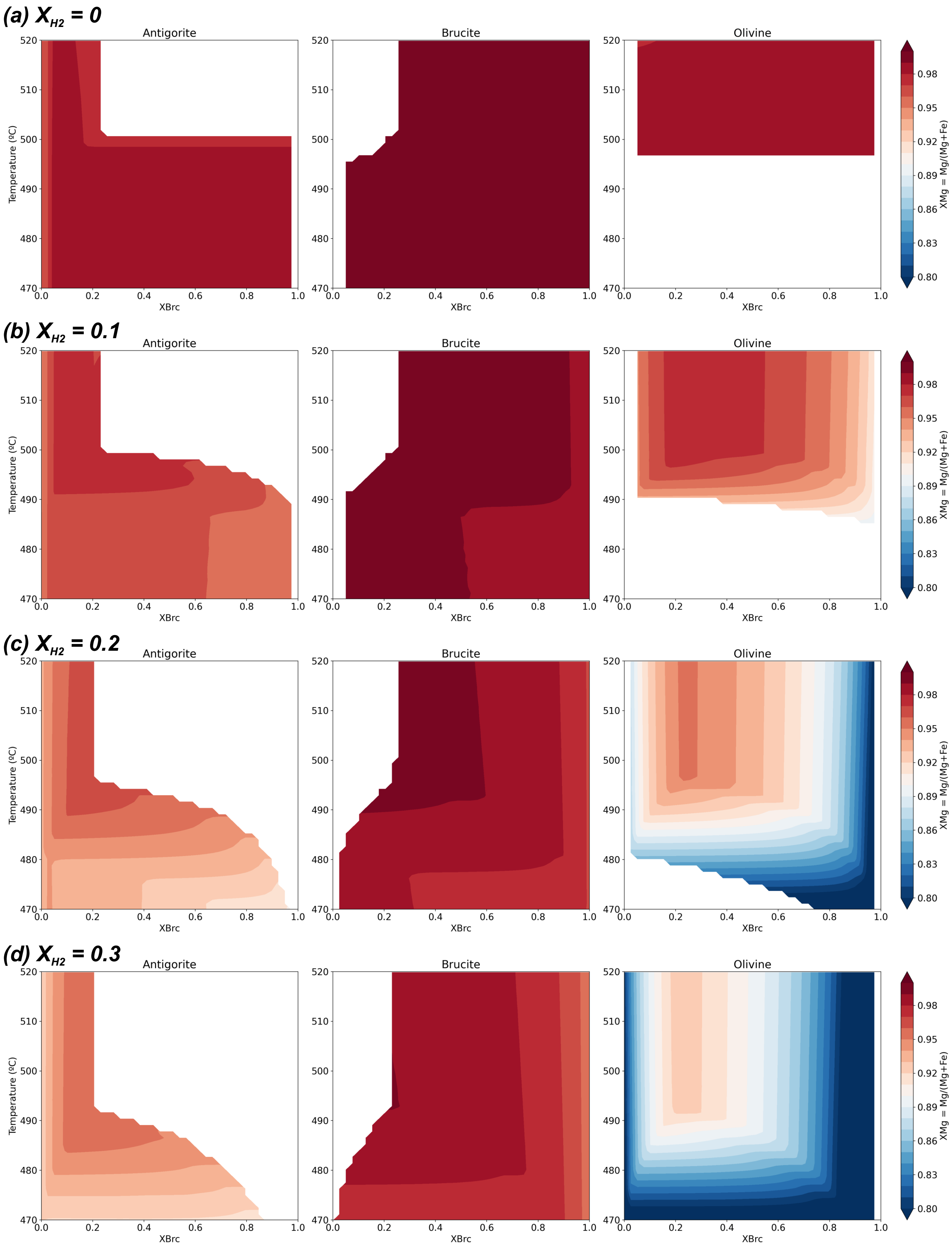

Supplementary Fig. S12. Predicted XMg compositions of antigorite, brucite and olivine as a function of the local effective equilibration volume‘s composition from serpentinite to brucite-vein (X_{Brc}) at different X_{H2} (c.f. Fig. 8).

E – Redox mass balance in the experiment sample

Fig. S13 shows the H_2 consumption (m(H_2), in mol/kg) in our experiment related to redox dehydration (reaction R3, main text), calculated for each point in the segmented EDX-map (Fig. 4a, main text) based on the Fe-content of olivine with $X_{Mg} < X_{Mg}^{Brc,Atg}$:

$$m(H_2) = \frac{2}{3} * \frac{(X_{Mg} * \rho_{Fo} + (1 - X_{Mg}) * \rho_{Fa})}{(X_{Mg} * M_{Fo} + (1 - X_{Mg}) * M_{Fa})} * X_{Mg} * \frac{1000}{\rho_{bulk}}. \qquad \text{(R-S6)}$$

X_{Mg} corresponds to Mg/(Mg + Fe^{2+}) in olivine, and ρ_{Fo} and ρ_{Fa} and M_{Fo}, M_{Fa} are the densities and molar masses of forsterite and fayalite endmembers, respectively. To exclude the Fe^{2+} fraction in olivine formed by redox-neutral brucite dehydration, the calculation was restricted to olivine with $X_{Mg} < X_{Mg}^{Brc,Atg}$, the latter being the average Mg/(Mg + Fe^{2+}) of brucite and antigorite in the starting material (0.985; see section "Starting material" in the main text). To obtain the same concentration units as used in the thermodynamic models (mol/kg), we normalize to the density of the bulk rock assemblage after dehydration (ρ_{bulk} = 2.58 g/cm^3, including fluid). The factor 2/3 derives from the stoichiometry of reaction R3.

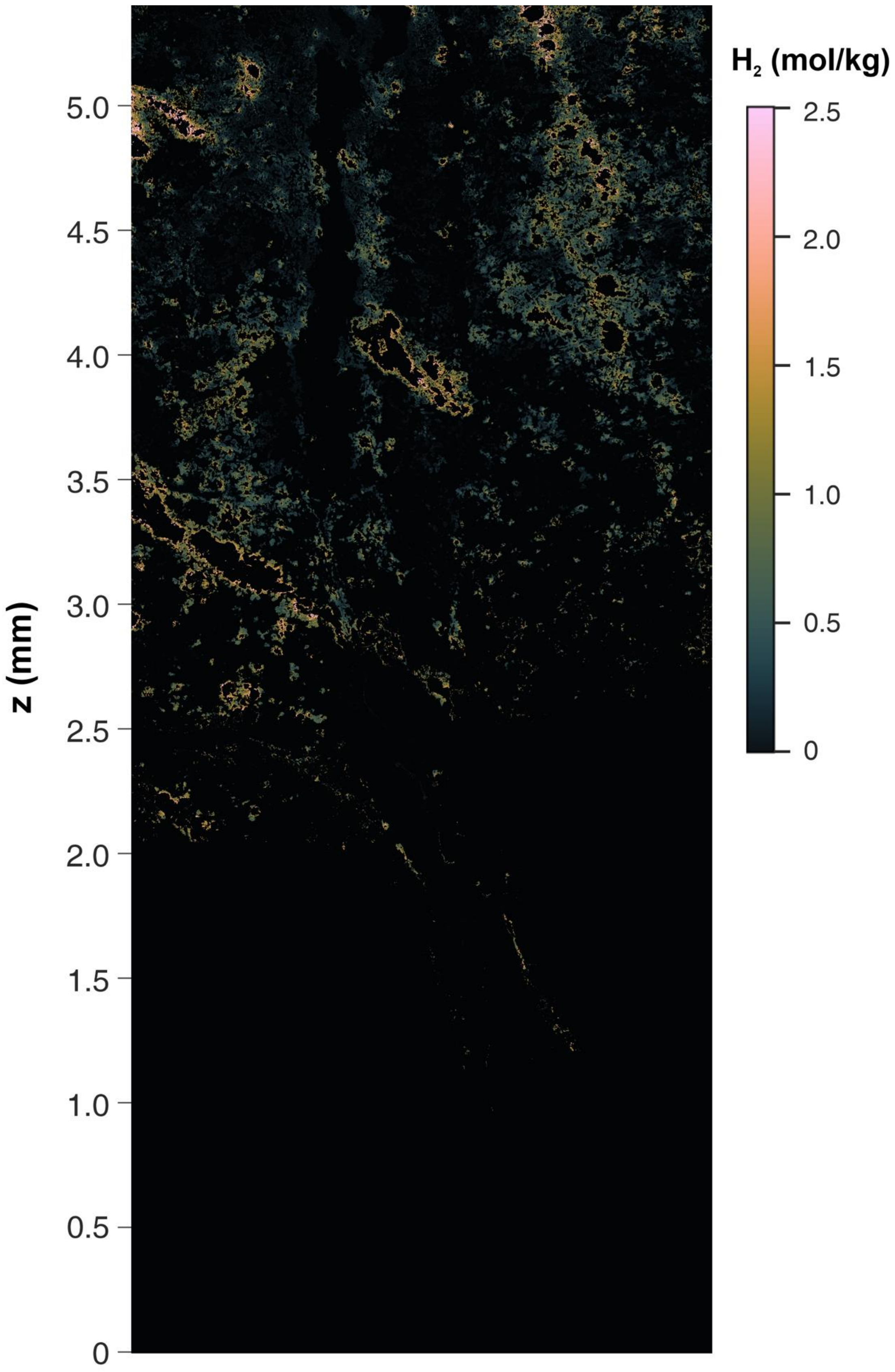

Figure S13: *H_2 consumption by redox dehydration in the experiment, based on the Fe-content in olivine derived from EDX measurements (c.f. Fig. 4a, main text).*

F –Discussion of H_2 production/ingress and oxygen fugacities in the experiment

The experimental sample was enclosed in a gold capsule, separated from the graphite heater by an MgO (periclase) spacer (Fig. S1). The MgO spacer comprises an overall volume 786 mm^3, corresponding to about 2.83 g or 0.07 mol MgO. This compares to a sample volume of 29.2 mm^3, with an estimated maximum fluid volume produced by dehydration of 2.19 mm^3 (< 15 vol% fluid in half of the sample volume, c.f. Figs. 2, 3, and 9). The latter corresponds to a total fluid mass of about 0.00224 g or 0.00012 mol (H_2O equivalent). The capsule was welded on the upper, high-temperature end and crimped on the lower-temperature end. Although gold deforms easily, allowing the capsule to seal upon compression, it is possible that a small quantity of the dehydration fluid left the capsule during the experiment at the crimped end. If coming into contact with the graphite heater, water could form COH-Fluid, including H_2. However, as stated in the main text, porosity produced by dehydration in the sample did not collapse (Fig. 3; Fig. 7), indicating that there was no major fluid loss out of the capsule. Furthermore, within the MgO spacer the dehydration fluid will react to brucite according to the reaction $H_2O + MgO = Mg(OH)_2$, since brucite is always more stable at the investigated P-T conditions (Mirwald, 2005). Considering the very fast brucite formation (e.g., Eberhard et al., 2022) and the three orders of magnitude higher abundance of MgO in the assembly compared to the dehydration fluid quantity, we do not expect dehydration fluid to react with the graphite heater.

The formation of Fe-rich olivine on the expense of magnetite and the trace appearance of iron (c.f., main text) require a reducing agent. Awaruite and similar Ni-Fe-alloys, which could produce H_2 during reaction to Ni-Fe sulfides and magnetite (Peretti et al., 1992), were not observed in the starting material. Based on the redox mass balance of the oxidized starting material ($Fe^{3+}/Fe_{total} = 0.73$) in comparison to the reducing capacity required for the observed Fe-bearing olivine quantity, internally-derived reducing agents are unlikely. We consider that H_2 must therefore have derived from the assembly and entered the capsule mostly by diffusion, although we do not exclude that some H_2 ingress may also have occurred via the crimped part of the gold capsule at the low-temperature end. H_2 ingress has been observed in various other subsolidus serpentinite experiments using graphite heaters (e.g., Eberhard et al., 2023; Iacovino et al., 2020; Merkulova et al., 2017). Two factors are decisive for such H_2 ingress into the capsule and the subsequent reduction of the sample: (i) the absolute quantities of reducing agents produced in the assembly and consumed by the sample, i.e., the *redox budgets* (reflected

by X_{H2} used in our models); and (ii) the difference in oxygen fugacities (f_{O2}) between the sample and the heater (which alternatively may be expressed as the difference in chemical potentials of oxygen or hydrogen), being the driving force of diffusion along a chemical gradient, i.e., the *redox potential*.

We infer that the H_2 source in our experiment is the graphite heater reacting with H_2O residual moisture in the assembly: The local bulk composition at the heater-MgO interface consists of periclase, graphite and moisture, resulting in the reaction $MgO + C + H_2O = Mg(OH)_2$ + COH-fluid, with MgO and C being massively in excess compared to a limited but unknown quantity of moisture. In such a system f_{O2} is not constrained but depends on the composition of the COH-fluid along the graphite-saturation curve (Fig. S14 a). The reaction consumes H_2O, and, in absence of an O_2 source, the water activity of the residual, graphite-saturated COH-fluid decreases in favor of $CH_4 + H_2$ (X_O decreases towards reducing fluid composition; *c.f.*, Connolly 1995; Golubkova et al., 2016). Eventually all H_2O will be consumed, and the fluid will be composed entirely of gaseous components H_2, CH_4, CO_2 and negligible CO (O_2 not being stable). At 500 °C and 1.5 GPa, this process decreases f_{O2} to log $f_{O2} < –27.4$ at $X_O < 0.01$ (Fig. S14 a & b). This is substantially lower than the f_{O2} of the initial starting material in the capsule (Fig. S14 c). Even a G-COH fluid at the water maximum ($X_O = 0.333$) exhibits low enough f_{O2} (ΔFMQ –0.5; Fig. S14 d) to induce a reduction equivalent to up to 0.18 mol/kg H_2 ingress (e.g., orange stars in Fig. S14 b, c & d). With progressive decrease of X_O of the G-COH fluid due to periclase hydration, f_{O2} of the graphite heater quickly attains values lower than the f_{O2} of magnetite-bearing assemblages in the serpentinite (yellow to green stars in Fig. S14 a – d; ΔFMQ << –3).
Even though G-COH fluids are predicted to contain higher CH_4 concentrations than H_2 at the experimental PT-conditions (see also Golubkova et al., 2016), methane molecules are too large for effective diffusion and the diffusive flux of H_2 towards domains with higher f_{O2} (i.e. the sample) will drive the continuous reaction $CH_4 = C + 2\ H_2$ at the graphite-heater. This process will lead to H_2 ingress into the capsule as long as (i) H_2 is produced at the heater, and (ii) there is an effective chemical potential gradient of H_2 (i.e., a higher f_{O2} in the sample compared to the G-COH fluid). This explains the observed H_2 ingress into the sample, driving Fe-olivine formation at the expense of magnetite.

Native iron was observed mostly within the brucite vein in the low-temperature part of the sample (Fig. 4c). Low enough f_{O2} for native iron formation may in principle be reached in the

assemblage when G-COH fluid composition drops below $X_O \approx 0.01$ (Fig. S14 c & d). However, it is still difficult to overall reach such low f_{O2} in the sample as this should in principle imply consumption of all magnetite. More likely, iron formation was due to local equilibrium effects: as shown in Figure S14 e, f_{O2} of the magnetite to iron transition (*IM-buffer*) is lower in Si-depleted compositions such as those representative of the interior of brucite veins. The same absolute quantity of H_2 ingress that produces the Fe-bearing olivine assemblages with remnant magnetite in the serpentinite matrix (e.g. $X_{H2} = 0.2$ in Fig. S14 d) can form locally stable native iron in brucite-dominated local vein compositions (Fig. S14 e). We infer therefore that the native iron present around some magnetite rims within the brucite vein in the low-temperature part of the sample is mainly due to local equilibrium effects, while the overall H_2 ingress is driven by the f_{O2} contrast between the bulk serpentinite sample and the heater assemblage as discussed above. The lack of re-equilibration between the very reducing, iron bearing domains at magnetite rims in brucite vein interiors and the oxidized antigorite–magnetite–brucite matrix surrounding the brucite vein is likely due to a lack of interconnected, aqueous-fluid filled porosity in the low-temperature part of the sample. In the high-temperature part of the sample, brucite dehydration resulted in the formation of a porous olivine-rich layer, facilitating re-equilibration. In this part of the sample we did not observe native iron.

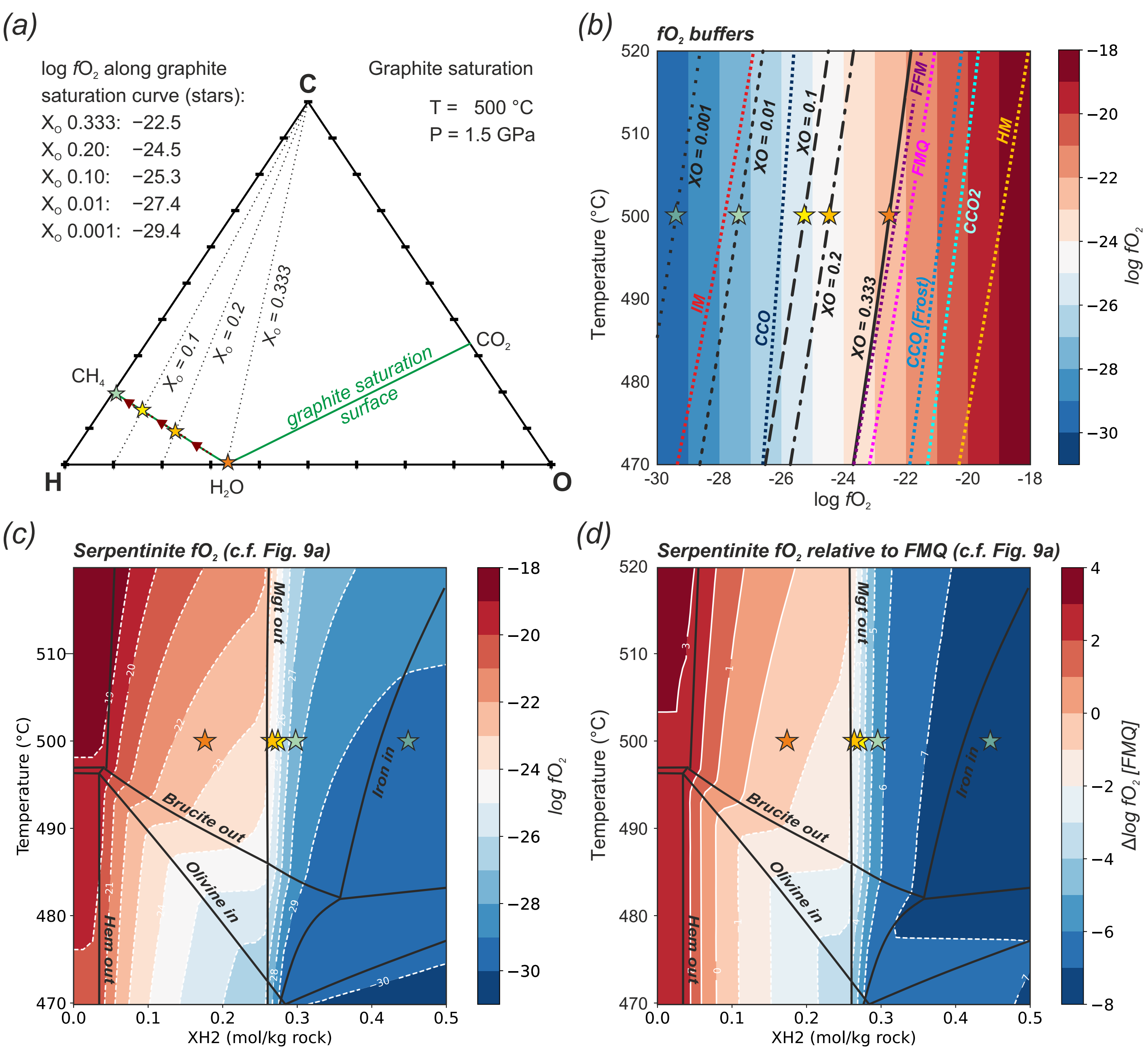

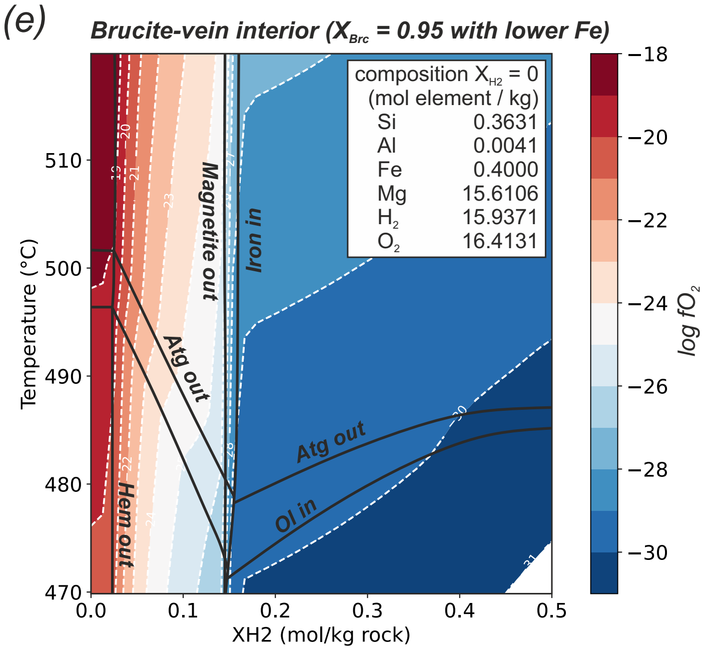

Fig. S14 Oxygen fugacities of the graphite heater and the sample. (a) compositions of graphite-saturated GCOH-fluids showing the evolution towards highly reducing fO_2 with decreasing X_O (calculated after Boutier et al., 2024) upon H_2O consumption due to MgO hydration (c.f. Golubkova et al., 2016). **(b)** logfO_2 of various oxygen buffers and GCOH-fluid with variable X_O. **(c & d)** logfO_2 and ΔlogfO_2[FMQ] of serpentinite with variable redox budget expressed as X_{H2} (same phase diagram as Fig. 9a). Stars show the reduction potential of the different GCOH fluids from a). **(e)** logfO_2 of a brucite-vein interior composition, showing that iron stability can locally be reached with the same absolute H_2 ingress as that recorded by the bulk serpentinite.

G – Additional supplementary figures

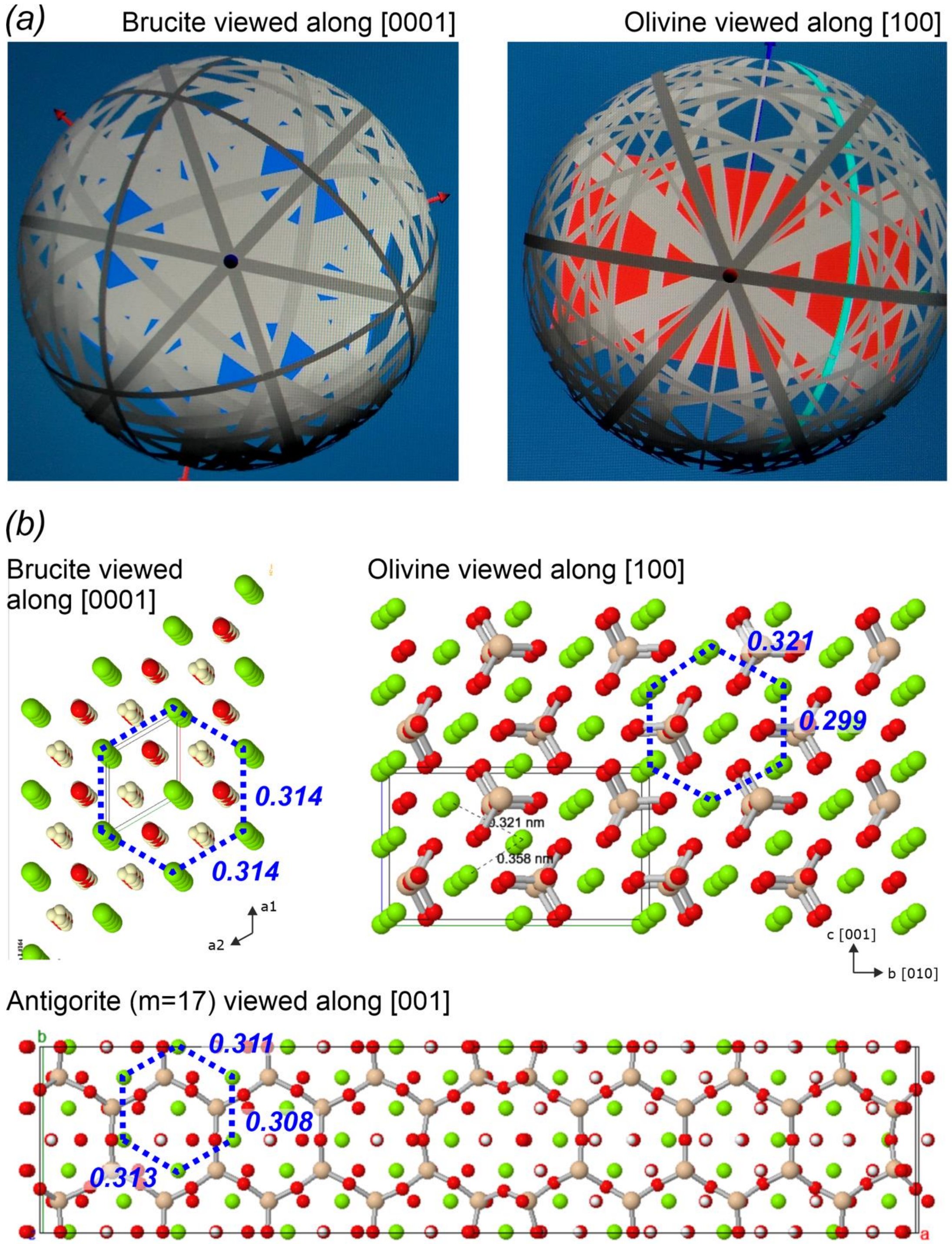

***Figure S15**: **(a)** predicted Kikuchi-patterns (AZTEC software, HKL and Inorganic crystal structure database) for brucite and olivine projected along [0001] and [100], respectively, showing that main bands in these orientations are similar and thus prone to misindexation. Misindexation artefacts can be reduced by improved band contrasts through good surface polishing, acquisition with low binning, and re-assignment of misindexed points based on simultaneous EDX data. **(b)** Crystal structure models of brucite, viewed along [0001], olivine along [100] and antigorite (polysome m=17) along [001], showing similar spacings between Mg-positions in these projection planes (blue values, in nm), and hexagonal (pseudo-) symmetry of all phases. Green: Mg; beige: Si; red: oxygen; white: hydrogen.*

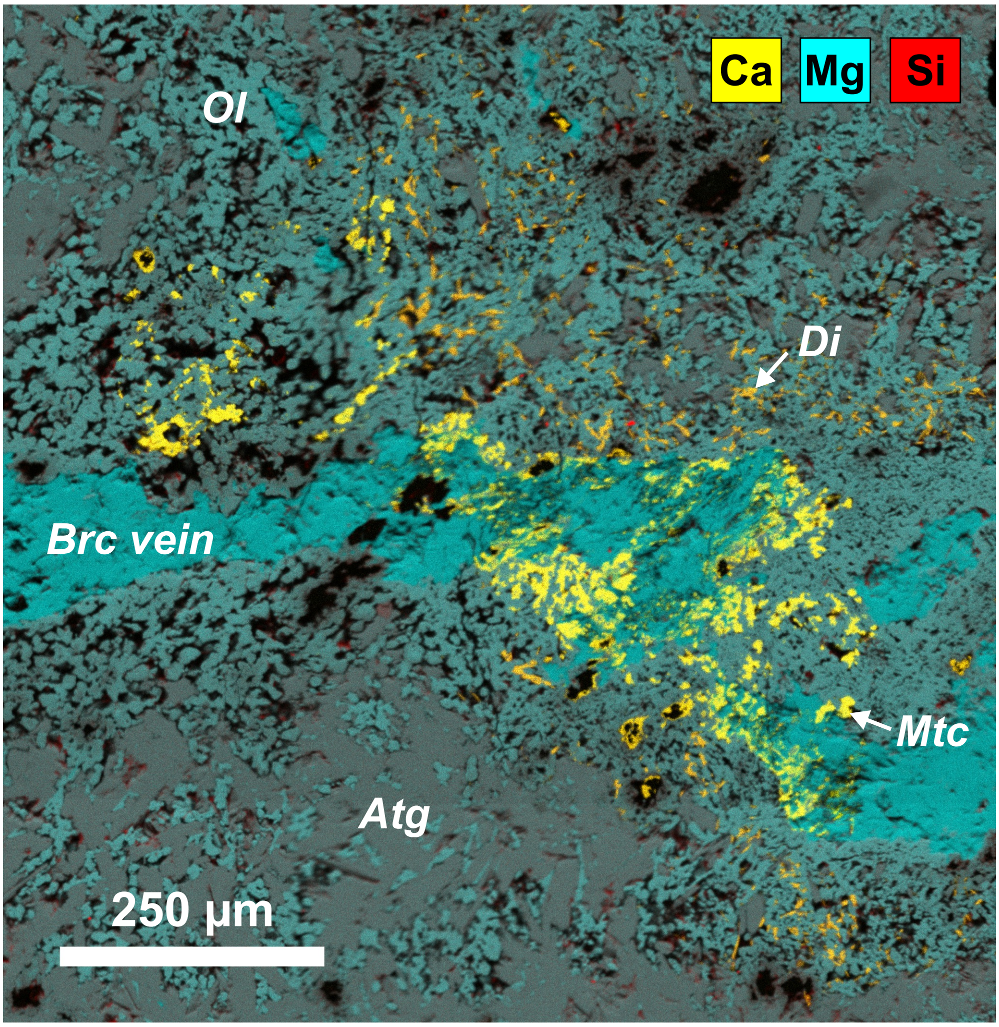

Fig. S16. *False-color EDX map part showing the detailed area in the experiment sample where monticellite (Mtc; yellow) and diopside (Di; orange) occur (c.f. Fig. 3a). Monticellite is primarily present in domains within the remnant brucite vein and inside olivine-pore aggregates, while diopside also occurs in contact with antigorite. This distribution reflects the increasing Si chemical potential from the remnant brucite vein towards the matrix. Monticellite formed inside the brucite vein due to locally high Ca chemical potential - likely due to former local presence of dolomite, clinopyroxene, or andradite - in combination with low Si in the local equilibration volume, and low f_{O2} imposed by H_2 ingress.*

References

Arblaster, J. W. (2016). Thermodynamic Properties of Gold. Journal of Phase Equilibria and Diffusion. Springer New York LLC 37, 229–245.

Boutier, A., Martinez, I., Daniel, I., Tumiati, S., Siron, G., Vitale Brovarone, A. (2024). Thermotopes-COH—A software for carbon isotope modeling and speciation of COH fluids. Computers & Geosciences 184, 105533.

Connolly, J.A.D. (1995). Phase diagram methods for graphitic rocks and application to the system C−O−H−FeO−TiO2−SiO2. Contributions to Mineralogy and Petrology 119, 94-116.

Eberhard, L., Frost, D.J., McCammon, C.A., Dolejš, D., Connolly, J.A.D. (2023). Experimental Constraints on the Ferric Fe Content and Oxygen Fugacity in Subducted Serpentinites. Journal of Petrology 64, egad069.

Eberhard, L., Thielmann, M., Eichheimer, P., Néri, A., Suzuki, A., Ohl, M., Fujita, W., Uesugi, K., Nakamura, M., Golabek, G.J., Frost, D.J. (2022). A New Method for Determining Fluid Flux at High Pressures Applied to the Dehydration of Serpentinites. Geochemistry, geophysics, geosystems 23, e2021GC010062.

Ghosh, S., Ghosh, A., Mukherjee, J. & Banerjee, R. (2015). Improved thermal properties of borosilicate glass composite containing single walled carbon nanotube bundles. RSC Advances. Royal Society of Chemistry 5, 51116–51121.

Golubkova, A., Schmidt, M.W., Connolly, J.A.D. (2016). Ultra-reducing conditions in average mantle peridotites and in podiform chromitites: a thermodynamic model for moissanite (SiC) formation. Contributions to Mineralogy and Petrology 171, 41.

Grizinvold, F., Stolen, S., Westrum, E. F., Labban, A. K. & Uhrens, B. (1988). Hear Capacity and Thermodynamic Properties of Ditungsten Carbide, W2C1-x, from 10 to 1000 K. Thermochimica Acta 129, 115–125.

Gummow, R. J. & Sigalas, I. (1988). The Thermal Conductivity of Talc as a Function of Pressure and Temperature. International Journal of Thermophysics 9, 1111–1120.

Hernlund, J., Leinenweber, K., Locke, D. & Tyburczy, J. A. (2006). A numerical model for steady-state temperature distributions in solid-medium high-pressure cell assemblies. American Mineralogist. Mineralogical Society of America 91, 295–305.

Holland, T. J. B. & Powell, R. (2011). An improved and extended internally consistent thermodynamic dataset for phases of petrological interest, involving a new equation of state for solids. Journal of Metamorphic Geology 29, 333–383.

Iacovino, K., Guild, M.R., Till, C.B. (2020). Aqueous fluids are effective oxidizing agents of the mantle in subduction zones. Contributions to Mineralogy and Petrology 175, 36.

Jiao, Y., Stillinger, F.H. & Torquato, S. (2007). Modeling heterogeneous materials via two-point correlation functions: Basic principles. Physical Review E—Statistical, Nonlinear, and Soft Matter Physics, 76. 031110.

Kanamori, H., Fujii, N. & Mizutani, H. (1968). Thermal diffusivity measurement of rock-forming minerals from 300° to 1100°K. Journal of Geophysical Research. American Geophysical Union (AGU) 73, 595–605.

Kanit, T., Forest, S., Galliet, I., Mounoury, V. & Jeulin, D. (2003). Determination of the size of the representative volume element for random composites: statistical and numerical approach. International Journal of solids and structures, 40. 3647-3679.

Lopes, P.C.F., Vianna, R.S., Sapucaia, V.W., Semeraro, F., Leiderman, R. & Pereira, A.M.B. (2023). Simulation toolkit for digital material characterization of large image-based microstructures. Computational Materials Science, 219. 112021.

Menzel, M.D., Garrido, C.J., López Sánchez-Vizcaíno, V., Marchesi, C., Hidas, K., Escayola, M.P., Delgado Huertas, A. (2018). Carbonation of mantle peridotite by CO_2-rich fluids: the formation of listvenites in the Advocate ophiolite complex (Newfoundland, Canada). Lithos 323, 238-261.

Merkulova, M.V., Muñoz, M., Brunet, F., Vidal, O., Hattori, K., Vantelon, D., Trcera, N., Huthwelker, T. (2017). Experimental insight into redox transfer by iron- and sulfur-bearing serpentinite dehydration in subduction zones. Earth and Planetary Science Letters 479, 133-143.

Mirwald, P.W. (2005). The fine-structure of the dehydration boundary of brucite (Mg(OH)2) up to 3.5 GPa - indication of anomalous PVT behaviour of supercritical H2O. European Journal of Mineralogy 17, 537-542.

Moarefvand, A., Gasc, J., Fauconnier, J., Baïsset, M., Burdette, E., Labrousse, L. & Schubnel, A. (2021). A new generation Griggs apparatus with active acoustic monitoring. Tectonophysics. Elsevier B.V. 816.

Osako, M., Yoneda, A. & Ito, E. (2010). Thermal diffusivity, thermal conductivity and heat capacity of serpentine (antigorite) under high pressure. Physics of the Earth and Planetary Interiors 183, 229–233.

Peretti, A., Dubessy, J., Mullis, J., Frost, B.R., Trommsdorff, V. (1992). Highly reducing conditions during Alpine metamorphism of the Malenco peridotite (Sondrio, northern

Italy) indicated by mineral paragenesis and H2 in fluid inclusions. Contributions to Mineralogy and Petrology 112, 329-340.

Powell, R. W., Ho, C. Y. & Liley, P. E. (1966). Thermal conductivity of Selected Minerals. National Standard Referece Data Series - National Bureau of Standards - 8.

Schindelin, J., Arganda-Carreras, Ignacio., Frise, E., Kaynig, V., Longair, M., Pietzsch, T., Preibisch, S., Rueden, C., Saalfeld, S., Schmid, B., & others (2012). Fiji: an open-source platform for biological-image analysis. Nature methods 9, 676 - 682.

Slifka, A. J., Filla, B. J. & Phelps, J. M. (1998). Thermal Conductivity of Magnesium Oxide from Absolute, Steady-State Measurements. Journal of Research of the National Institute of Standards and Technology. National Institute of Standards and Technology 103, 357–363.

Torquato, S., 2002. Random Heterogeneous Materials: Microstructure and Macroscopic Properties. Springer Science+Business Media New York 2002.

Wattanasarn, H. & Seetawan, T. (2013). Studies thermophysical properties of MgO by first principle simulation. Advanced Materials Research, 139–143.

Williams, W. S. (1998). The Thermal Conductivity of Metallic Ceramics. Thermal Management 62–66.

Yeong, C.L.Y. & Torquato, S. (1998). Reconstructing random media. Physical review E, 57. 495.
